# Cisd2 is essential to delaying cardiac aging and to maintaining heart functions

**DOI:** 10.1371/journal.pbio.3000508

**Published:** 2019-10-08

**Authors:** Chi-Hsiao Yeh, Zhao-Qing Shen, Shao-Yu Hsiung, Pei-Chun Wu, Yuan-Chi Teng, Yi-Ju Chou, Su-Wen Fang, Chian-Feng Chen, Yu-Ting Yan, Lung-Sen Kao, Cheng-Heng Kao, Ting-Fen Tsai

**Affiliations:** 1 Department of Thoracic and Cardiovascular Surgery, Chang Gung Memorial Hospital, Keelung, Taiwan; 2 College of Medicine, Chang Gung University, Taoyuan, Taiwan; 3 Department of Life Sciences and Institute of Genome Sciences, National Yang-Ming University, Taipei, Taiwan; 4 Program in Molecular Medicine, School of Life Sciences, National Yang-Ming University and Academia Sinica, Taipei, Taiwan; 5 Brain Research Center, National Yang-Ming University, Taipei, Taiwan; 6 Genome Research Center, National Yang-Ming University, Taipei, Taiwan; 7 Institute of Biomedical Sciences, Academia Sinica, Taipei, Taiwan; 8 Center of General Education, Chang Gung University, Taoyuan, Taiwan; 9 Aging and Health Research Center, National Yang-Ming University, Taipei, Taiwan; 10 Institute of Molecular and Genomic Medicine, National Health Research Institutes, Zhunan, Taiwan; University of Cincinatti, UNITED STATES

## Abstract

CDGSH iron-sulfur domain-containing protein 2 (Cisd2) is pivotal to mitochondrial integrity and intracellular Ca^2+^ homeostasis. In the heart of *Cisd2* knockout mice, Cisd2 deficiency causes intercalated disc defects and leads to degeneration of the mitochondria and sarcomeres, thereby impairing its electromechanical functioning. Furthermore, Cisd2 deficiency disrupts Ca^2+^ homeostasis via dysregulation of sarco/endoplasmic reticulum Ca^2+^-ATPase (Serca2a) activity, resulting in an increased level of basal cytosolic Ca^2+^ and mitochondrial Ca^2+^ overload in cardiomyocytes. Most strikingly, in *Cisd2* transgenic mice, a persistently high level of Cisd2 is sufficient to delay cardiac aging and attenuate age-related structural defects and functional decline. In addition, it results in a younger cardiac transcriptome pattern during old age. Our findings indicate that Cisd2 plays an essential role in cardiac aging and in the heart’s electromechanical functioning. They highlight Cisd2 as a novel drug target when developing therapies to delay cardiac aging and ameliorate age-related cardiac dysfunction.

## Abstract 2

Cardiomyocytes from young mice lacking the mitochondrial iron-sulfur domain-containing protein Cisd2 show signs of premature aging; conversely, cardiomyocytes from old transgenic mice with a persistently high level of Cisd2 appear “young.” This highlights Cisd2 as a novel drug target when developing therapies to delay cardiac aging.

## Introduction

As human life span progressively increases globally, knowledge of the effects of aging on human pathophysiology has become of great relevance to medicine [1]. By 2050, the world’s population aged 60 years and older is expected to reach 2 billion, up from 900 million in 2015 [2]. Cardiovascular disease (CVD) is the leading cause of death globally and accounts for 40% of all deaths among the aged group [3]. Aging is one of the most important factors that has a significant impact on the cardiovascular system. However, there remain many unanswered questions regarding CVD or cardiac function during life span shortening (premature aging) or life span extension (longevity) studies [3]. Indeed, the interplay between aging and age-related cardiac dysfunction is not completely understood.

The CDGSH iron-sulfur domain-containing protein 2 (CISD2) is an evolutionarily conserved gene encoding a small protein (135 amino acids). It contains a transmembrane domain at the N terminal and a single CDGSH domain at the C terminal, which harbors a redox-active 2Fe-2S cluster. CISD2 forms a homodimer and is present in a range of subcellular locations, particularly the endoplasmic reticulum (ER), the mitochondrial outer membrane (MOM), and the mitochondria-associated ER membranes (MAMs) [4]. CISD2’s subcellular localization suggests it plays a crucial role in physiological control. Cisd2 is essential to mitochondrial integrity and intracellular Ca^2+^ homeostasis [5–9]. In Cisd2 knockout (KO) mice, Cisd2 deficiency shortens life span and drives premature aging [10], whereas in Cisd2 transgenic (TG) mice, a persistently high level of Cisd2 extends healthy life span. Cisd2 ameliorates age-associated degeneration of skeletal muscles and neurons, protects mitochondria from age-associated damage and functional decline, and attenuates the age-associated reduction that affects whole-body energy metabolism [11].

Dysregulation of Ca^2+^ homeostasis within cardiomyocytes is one of the traits of heart failure [12,13]. Large amounts of Ca^2+^ are released from the sarcoplasmic reticulum (SR) into the cytosol to cause cardiomyocytic contraction. Upon signal termination, cardiomyocytic relaxation is initiated by Ca^2+^ ATPase (sarco/endoplasmic reticulum Ca^2+^-ATPase [SERCA2]), which uptakes cytosolic Ca^2+^ into the SR, thus restoring cytosolic Ca^2+^ to a basal level [14]. Cardiomyocytes isolated from failing hearts show a prolonged and slow Ca^2+^ transient and poor Ca^2+^ removal performance during the diastolic phase [15]; this results in a persistent elevation of Ca^2+^ in the cytosol. One consequence of this is likely to be Ca^2+^ overload of the mitochondria leading to reduced ATP production and increased reactive oxygen species (ROS) levels. This in turn causes cardiomyocytic injury, heart failure, and eventually death [16,17]. Currently, the classical approaches widely used to treat adult hearts, such as ischemic preconditioning and postconditioning [18], are less effective when used on aged hearts [19]. Accordingly, novel strategies based on new therapeutic targets are required to retard cardiovascular aging and attenuate CVD among the elderly.

Importantly, CISD2 is the causative gene for Wolfram syndrome 2 (WFS2; MIM 604928). WFS (MIM 222300), which is an autosomal recessive disease associated with neurodegeneration and metabolic disorders. The clinical manifestations of WFS are highly variable and include diabetes insipidus, diabetes mellitus, optic atrophy, and deafness; thus, it is also known as DIDMOAD syndrome. Currently, two disease genes have been identified, WFS1 and CISD2, which cause WFS1 and WFS2, respectively [6,7]. Intriguingly, cardiac abnormalities have been reported to affect 16.1% of WFS patients; these patients have congenital ventricular septal defects, systolic ejection murmurs, and/or abnormal echocardiography [20]. These cardiac defects may participate in the morbidity and mortality of WFS. However, the pathogenic basis of these abnormalities is not known, and the underlying mechanisms remain unresolved.

Here, we use four mouse models and one cell platform—namely, adult cardiomyocytes isolated from wild-type (WT), Cisd2KO, and Cisd2TG mice—to investigate the role of Cisd2 in cardiac function and explore the biological relevance of Cisd2 to cardiac aging. The four male mouse models are (1) young WT mice at 3 months old (3M) and 6 months old (6M), (2) prematurely aged Cisd2KO mice at 3M and 6M, (3) naturally aged WT mice at 26 months old (26M), and (4) long-lived Cisd2TG mice at 26M. The mean life span of these mouse models in our animal facility are Cisd2KO (19.04 ± 1.21 months, *n* = 27), WT (25.74 ± 0.62 months, *n* = 40), and Cisd2TG (30.72 ± 1.18 months, *n* = 34).

## Results

### Structural appearance of the hearts of naturally aged WT mice and prematurely aged Cisd2KO mice

**Fig 1:**
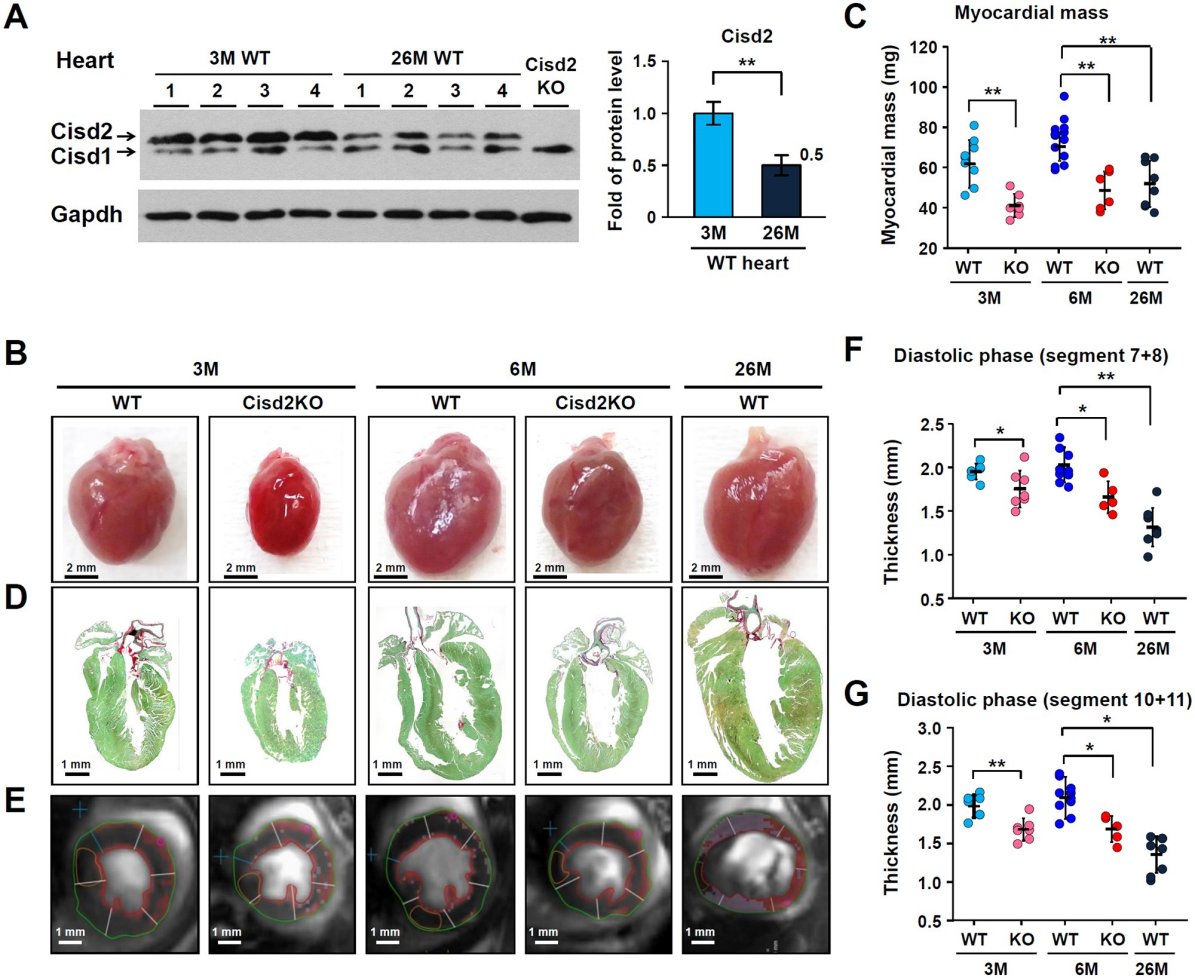
Down-regulation of Cisd2 in the naturally aged hearts and the cardiac pathology present in Cisd2KO and naturally aged WT mice. (A) Western blot analysis of myocardial Cisd2 protein levels using heart tissue samples obtained from young (3M) and old (26M) WT mice (*n* = 4). Gapdh was used as an internal control. (B) Representative photographs of hearts obtained from WT and Cisd2KO mice at 3M and 6M, as well as those obtained from old WT mice at 26M. Scale bar, 2 mm. (C) Myocardial weights of the LV (*n* ≥ 5 per group). (D) Long-axis low-power section of Sirius Red/Fast Green staining of the hearts with the aim of detecting collagen. Scale bar, 1 mm. (E) Representative MRI (7-T MRI) of a separate cohort of mice (*n* ≥ 5 per group) is presented. (F and G) Analysis of the MRI of diastolic segment 7+8 (anterior LV free wall, F) and diastolic segment 10+11 (interventricular LV wall, G). A significant decrease in left ventricular wall thickness can be noted in the old WT mice at 26M and in the Cisd2KO mice at 3M and 6M. The data are presented as mean ± SD and are analyzed by Student *t* test. *p < 0.05; **p < 0.005. All the mice used in this study are males. Values for each data point can be found in S1 Data. 3M, 3 months old; 6M, 6 months old; 26M, 26 months old; Cisd, CDGSH iron-sulfur domain-containing protein; Gapdh, glyceraldehyde 3-phosphate dehydrogenase; KO, knockout; LV, left ventricle; MRI, magnetic resonance imaging; WT, wild type.

The prematurely aged Cisd2KO mice as young animals display a panel of accelerated aging phenotypes that resemble the phenotypes of an old animal [10]. Additionally, the levels of Cisd2 have been shown to decrease in an age-dependent manner in various tissues of WT mice, such as skeletal muscle, during aging; interestingly, Cisd2 KO also leads to skeletal muscle degeneration [10,11]. Here, we investigated whether expression level of Cisd2 is reduced in the myocardium of naturally aged mice at 26M compared with young mice at 3M. The Cisd2 level was significantly reduced by about 50% in the myocardium of 26M mice compared with 3M mice (Fig 1A). This suggests that the reduced Cisd2 level might have an impact on age-related alterations that affect cardiac functioning.

In Cisd2KO mice, their hearts are smaller compared with age-matched, sex-matched controls (Fig 1B). However, there are no gross anatomical changes in the hearts of Cisd2KO mice, including the dimensions of the atrial and ventricular chambers by cardiac magnetic resonance imaging (MRI). Nevertheless, the myocardial weight of the left ventricle (LV) is reduced in the Cisd2KO and naturally aged WT mice (Fig 1C; S1A and S1B Fig). Sirius Red staining reveals an increased accumulation of collagen fibers (interstitial fibrosis) in the myocardium of Cisd2KO and naturally aged mice (Fig 1D and S1C Fig). Furthermore, MRI reveals a significant decrease in the LV wall thickness of Cisd2KO and naturally aged mice (Fig 1E–1G), which is similar to the decrease of LV mass in men with aging [21]. These results suggest that Cisd2 is required for maintenance of normal cardiac structure.

### Cisd2 deficiency causes cardiac electromechanical dysfunction

**Fig 2:**
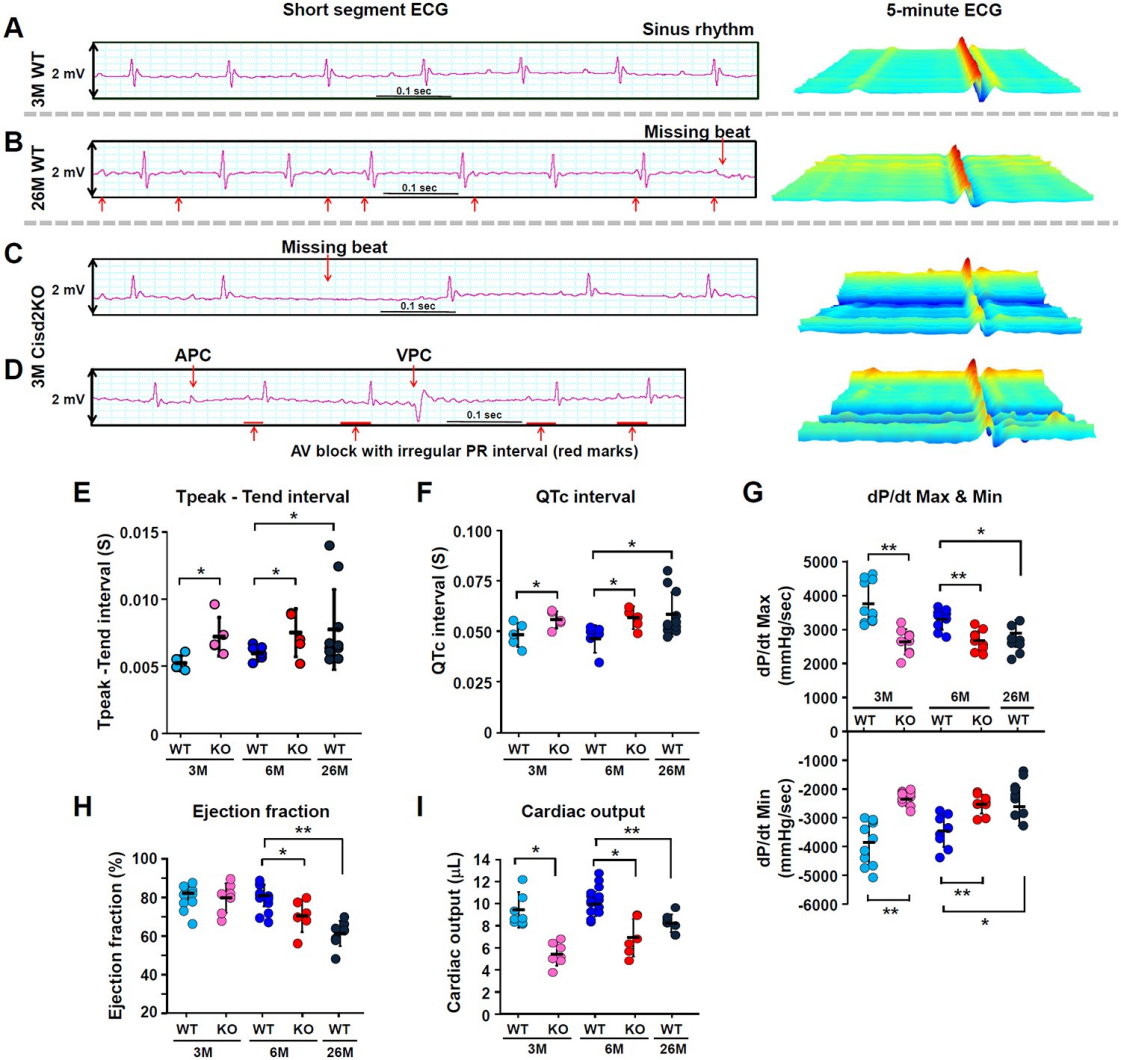
Cisd2KO mice exhibit cardiac electromechanical dysfunctions that are similar to those observed in the naturally aged WT mice. (A-D) Representative ECG tracings and continuous 5-minute waterfall plots recorded following anesthesia of the mice. A normal sinus rhythm ECG recorded from a young (3M) WT mouse (A). The irregular PR interval and missing beats recorded from an aged (26M) WT mouse (B). Representative dysrhythmic ECGs, namely a missing beat (C), AV block with irregular and progressive prolongation of PR interval, APCs, and VPCs (D), which were found in the Cisd2KO mice at 3M. (E and F) Measurements of the Tpeak–Tend intervals (E) and the QTc intervals (F) were obtained from 5 minutes of sequential beats obtained from whole ECG tracings. (G) Peak developed pressures and rates of pressure increase and rates of pressure decrease during left ventricular contraction; these were measured by Millar’s catheter in the mice. The dP/dt max is the maximal rate of pressure development, and the dP/dt min is the maximal rate of decay of pressure. The WT mice exhibited an age-dependent progression with respect to cardiac dysfunction with a reduced dP/dt Max and dP/dt min. (H and I) Left ventricular ejection fraction (H) and cardiac output (I) were measured and quantified by cardiac MRI. Data are presented as mean ± SD and are analyzed by Student *t* test. *p < 0.05; **p < 0.005. Mouse number *n* ≥ 5 for each group. Values for each data point can be found in S1 Data. 3M, 3 months old; 6M, 6 months old; 26M, 26 months old; APC, atrial premature complex; AV, atrioventricular; Cisd2KO, CDGSH iron-sulfur domain-containing protein 2 KO; ECG, electrocardiography; KO, knockout; MRI, magnetic resonance imaging; QTc, corrected QT; VPC, ventricular premature complex; WT, wild type.

Cisd2KO mice are viable and display no overt phenotype at birth. However, Cisd2KO mice have a shortened life span with 40% mortality by 10 months old (10M), with their survival beginning to decline rapidly after 5 months [10]. There is no significant difference in the following measurements between Cisd2KO and WT mice at 3M: heart rate and blood pressure (S1D–S1F Fig), as well as various serum tests including T_3_ and T_4_thyroid hormones, serum markers related to renal function, and serum markers related to lipid metabolism, blood glucose, insulin levels, and various electrolyte levels (S2 Fig). Although it is a challenge to identify differences of a few milliseconds in the electrocardiography (ECG) analysis of mice, it was noted that, in Cisd2KO mice, 5-minute longitudinal ECG analysis reveals at 3M the presence of atrial and ventricular premature contractions with atrioventricular block (Fig 2A–2D), together with prolongations of the Tpeak–Tend interval and the corrected QT (QTc) interval (Fig 2E and 2F; other ECG measurements are provided in S1 Table). Moreover, in Cisd2KO mice, cardiac MRI reveals that their hearts display a variety of contractile dysfunctions. There is a significant reduction in LV peak systolic pressure, namely reduced rates of LV pressure increase (dP/dt max) and pressure decrease (dP/dt min) (Fig 2G). Additionally, there is a significant reduction in the LV ejection fraction at 6M (Fig 2H) and in the cardiac output of 3M and 6M Cisd2KO mice (Fig 2I). In naturally aged mice at 26M, their cardiac performance, including both electrical (Fig 2E and 2F) and mechanical (Fig 2G–2I) aspects, resembles Cisd2KO mice at 6M. Taken together, these ECG and MRI findings reveal that Cisd2 is an essential protein for maintaining normal heart electrophysiological activity and cardiac contraction.

### Cisd2 deficiency causes intercalated disc defects and leads to degeneration of the mitochondria and sarcomeres in cardiac muscle

The intercalated disc is an indispensable structure that connects individual cardiomyocytes and allows them to work as a single functional unit via the synchronized contraction of cardiac muscle. To define the structural basis of the abnormal cardiac function caused by Cisd2 deficiency, we performed immunofluorescence (IF) staining and ultrastructural analysis to examine the integrity of the three types of cell junction that make up the intercalated disc, namely gap junctions, desmosomes, and fascia adherens.

**Fig 3:**
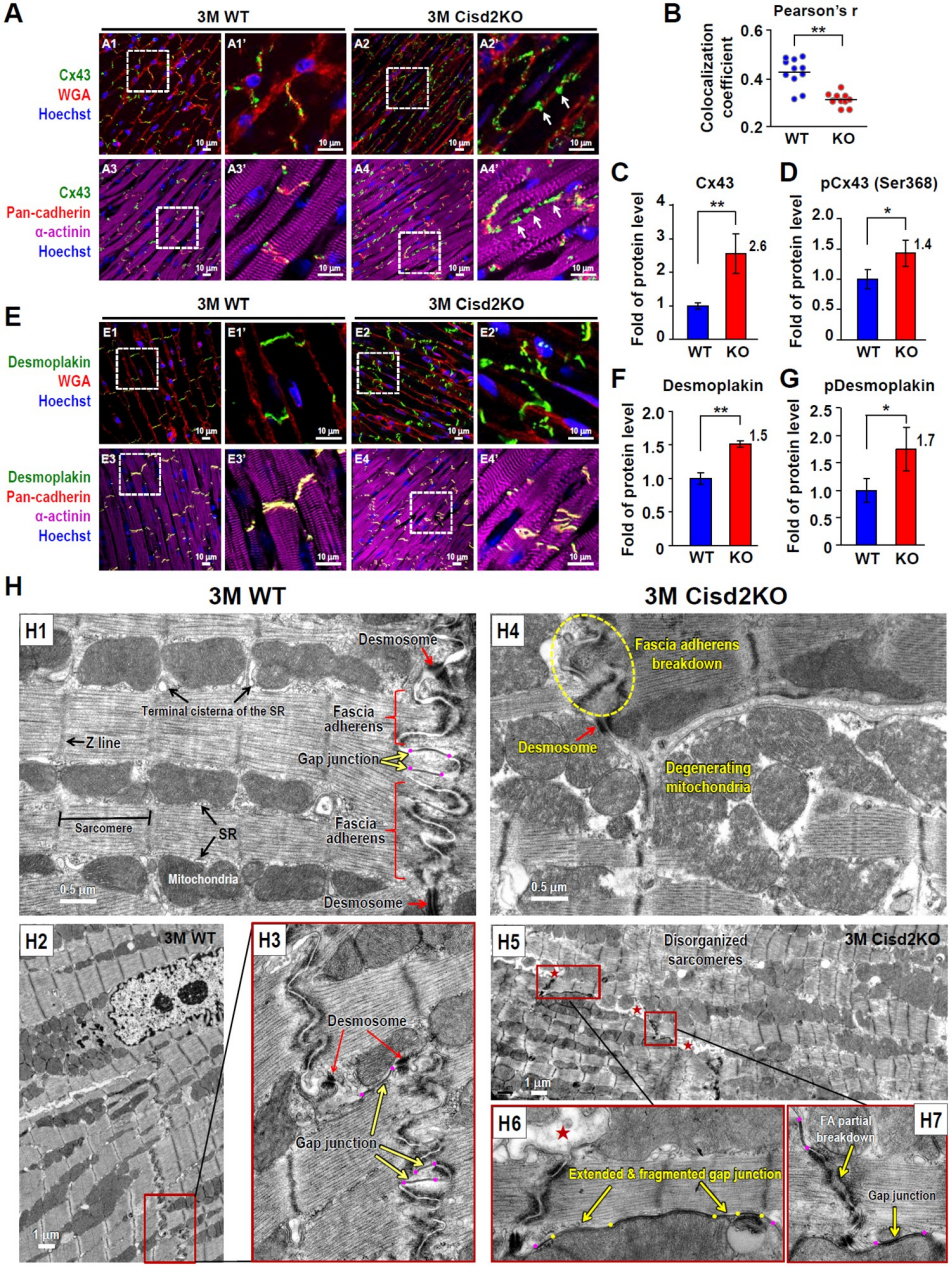
Cisd2 deficiency causes defects in the intercalated disc and leads to ultrastructural abnormalities in the cardiac muscle. (A) Lateralization of gap junctions in the cardiac muscle of Cisd2KO mice. Representative IF images of heart sections stained with antibodies against Cx43 (green) in order to localize gap junctions, against pan-cadherin (red) to localize the intercalated discs, and against α-actinin (purple) to stain muscle fibers. The sections were also stained with Hoechst (blue) to identify nuclei. White arrows indicate lateralization of gap junctions. (B) Colocalization coefficient of gap junction protein (Cx43) and intercalated disc protein (pan-cadherin) was analyzed by Pearson’s correlation. The computed value is presented as the Cx43/pan-cadherin colocalization coefficient. Data were collected from 10 randomly selected fields for each heart sample. There are at least three individual mice in each group. (C and D) Relative protein levels of Cx43 (C) and phosphorylated Cx43 (Ser368) (D) in the hearts of WT and Cisd2KO mice were analyzed by western blotting and then quantified (*n* = 4). (E) Maldistribution of desmosomes in the cardiac muscle of Cisd2KO mice. Representative IF images of heart sections stained with antibodies against desmoplakin (green), which is localized within desmosomes, with WGA (red) to stain cell membranes by binding to membrane glycoproteins, with antibodies against pan-cadherin (red) to localize the intercalated discs, and with antibodies against α-actinin (purple) to stain muscle fibers. The sections were also stained with Hoechst (blue) to identify nuclei. (F and G) Relative protein levels of desmoplakin (F) and phosphorylated desmoplakin (G) in the hearts of WT and Cisd2KO mice were analyzed by western blotting and then quantified (*n* = 4). (H) TEM analysis reveals ultrastructural defects in the intercalated discs of Cisd2KO cardiac muscle. Overt ultrastructural abnormalities (namely, shortening and breaking down of the FA; degeneration of the mitochondria, including mitochondria with ruptured outer and inner membranes and swollen mitochondria with fewer crista; and extension and fragmentation of gap junctions) were easily found in the cardiac muscle of Cisd2KO mice at 3M. In H5 and H6, red stars (★) indicate that parts of the space between the two membranes of the intercalated discs are expanded. In H3, H6, and H7, purple dots indicate the two ends of gap junctions. In H6, yellow dots and arrows indicate several degenerating regions with loss of compactness interlaced into an extended gap junction (between two purple dots). Data are presented as mean ± SD and are analyzed by Student *t* test. *p < 0.05; **p < 0.005. Values for each data point can be found in S1 Data. 3M, 3 months old; Cisd2, CDGSH iron-sulfur domain-containing protein 2; Cx43, Connexin 43; FA, fascia adherens; IF, immunofluorescence; KO, knockout; SR, sarcoplasmic reticulum; TEM, transmission electron microscopy; TG, transgenic; WGA, wheat germ agglutinin; WT, wild type.

Normally, the gap junctions allow action potentials to spread by permitting the passage of ions between cardiomyocytes, which produces depolarization of the cardiac muscle. Strikingly, IF staining of Connexin 43 (Cx43, a gap junction protein) reveals that Cisd2 deficiency results in the amplification and lateralization of gap junctions (Fig 3A). Extensive Cx43 staining was observed along the lateral borders of the cardiomyocytes in Cisd2KO mice; this abnormal pattern of lateralization of the gap junctions has been shown to be associated with a variety of heart diseases [22]. Furthermore, the colocalization coefficient of Cx43 and pan-cadherin, an intercalated disc protein, is significantly decreased in Cisd2KO mice, suggesting that a significant portion of lateralized gap junctions are not colocalized with the intercalated discs (Fig 3B). Moreover, the protein levels of Cx43 and phosphorylated Cx43 (Ser368) are significantly increased in Cisd2KO mice (Fig 3C and 3D; S3A and S3B Fig); this is consistent with the observation that elongation of the gap junctions is associated with degeneration into fragments, which occurs in addition to the lateralization when Cisd2 is absent. Intriguingly, a maldistribution of desmosomes in the cardiac muscle of Cisd2KO mice is detected by IF staining of desmoplakin, a desmosomal protein (Fig 3E). Western blotting reveals that the protein levels of desmoplakin and phosphorylated desmoplakin are significantly elevated in Cisd2KO mice (Fig 3F and 3G; S3C and S3D Fig). Since desmosomes join cells together by binding intermediate filaments during contraction, such a maldistribution is likely to contribute to the mechanical dysfunction and dysrhythmia of the Cisd2KO heart. No significant differences in the levels of other intercalated disc proteins (vinculin, cadherin, and alpha-actinin) were detected (S3E and S3F Fig).

To examine cell–cell junctions, organelle integrity, and myofibril organization at the ultrastructural level, we carried out transmission electron microscopy (TEM) analysis. In 3M WT mice (Fig 3HH1–3HH3), three types of cell junction were easily identified. A folded membrane was interwoven with the membrane of neighboring cardiomyocytes to form the fascia adherens, providing anchoring sites for actin; these are connected to the closest sarcomere. In 3M Cisd2KO mice (Fig 3HH4–3HH7), severe ultrastructural alterations were detected. These include breakdown of the fascia adherens, extension and fragmentation of the gap junctions, and partial degeneration of the desmosomes. Notably, areas in the space between the two membranes of the intercalated disc were frequently found to be expanded (Fig 3HH5 and 3HH6). Additionally, degenerated and swollen mitochondria, as well as disorganized and degenerated myofibrils, were easily detected in Cisd2KO mice. The severity of these ultrastructural abnormalities was even more obvious in the 6M Cisd2KO mice (S4 Fig). Collectively, the IF and TEM results reveal that Cisd2 plays an essential role in maintaining the integrity of the intercalated discs, the mitochondria, and the myofibrils of cardiac muscle.

### Cisd2 deficiency disrupts Ca^2+^ homeostasis via a dysregulation of Serca2a activity and results in mitochondrial Ca^2+^ overload of the adult cardiomyocytes

**Fig 4:**
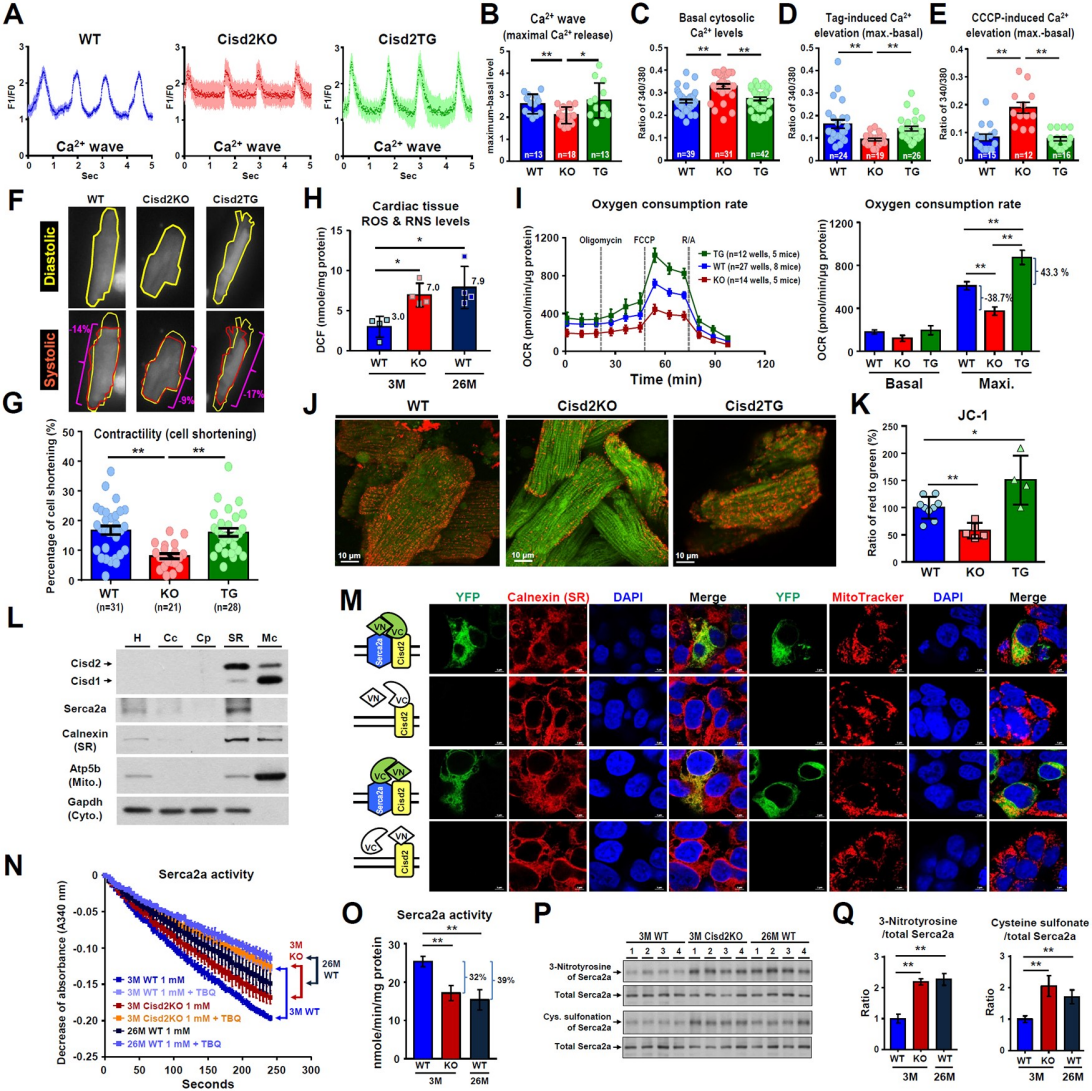
Cisd2 deficiency disrupts Ca^2+^ homeostasis via a dysregulation of Serca2a activity and this then results in mitochondrial Ca^2+^ overload and dysfunction in cardiomyocytes. (A and B) Spontaneous Ca^2+^ waves of beating cardiomyocytes isolated from WT, Cisd2KO, and Cisd2TG mice at 3M were measured by confocal microscopy (100 measures per second) using Fluo-4 AM staining. (A) The Ca^2+^ waves (F1/F0: fluorescence-intensity change normalized against the background fluorescence). Representative videos are provided in the supplemental information. (B) Quantification of maximal Ca^2+^ release (maximum-basal level). The data are presented as mean ± SD for 13–18 cells from 3–5 mice of each genotype. (C-E) The levels of cytosolic Ca^2+^ in single adult cardiomyocytes isolated from WT, Cisd2KO, and Cisd2TG mice were measured by fluorescence microscopy using Fura-2/AM staining. After measuring the basal level of cytosolic Ca^2+^ (first 50 seconds), Tag was added to release Ca^2+^ from the SR. Alternatively, CCCP was added to release Ca^2+^ from the mitochondria. Quantification of the basal cytosolic Ca^2+^ levels (C), Tag-induced Ca^2+^ elevation from the SR (D), and CCCP-induced Ca^2+^ elevation from mitochondria (E) of the adult cardiomyocytes. The data are presented as mean ± SEM for 12–42 cells from 5–6 mice of each genotype. (F) Contractility (cell shortening) was measured during the spontaneous beating of cardiomyocytes (representative videos are provided in the supplemental information). (G) Quantification of cell shortening for 21–31 cells from 5–6 mice of each genotype. The data are presented as mean ± SEM. (H) Quantification of ROS and RNS levels in the heart by measuring DCF levels using cardiac tissue samples (*n* = 4 for each group). The data are presented as mean ± SD. (I) OCRs of the isolated adult cardiomyocytes from different genotypes. The indicated chemicals (oligomycin A, FCCP, R/A) were added sequentially to determine the ATP-coupled respiration rate, the maximal respiration rate (Max), and the nonmitochondrial respiration rate, respectively, using a Seahorse XFe24 analyzer. (J and K) Representative confocal microscopic pictures (J) and quantification of JC-1 flowcytometric staining results (K), which were used to measure the mitochondrial membrane potential of adult cardiomyocytes isolated from WT, Cisd2KO, and Cisd2TG mice. (L) Western blot analysis of Cisd2, Serca2, Calnexin (SR), Atp5b (mitochondria), and Gapdh (cytosol) using different subcellular fractions prepared from the hearts of WT mice. A total of 10 μg of protein from each fraction was loaded. (M) Bimolecular fluorescence complementation assay using split-YFP constructs of Cisd2 and Serca2a. Cisd2 and Serca2a were fused with the C-terminal (VC) or N-terminal (VN) domain of the YFP protein and transfected into HEK-293T cells. The localization of the refolded Venus protein (green), SR (red) or mitochondria (red), and the cell nucleus (blue) are detected by confocal microscopy. Scale bar, 5 μm. (N) Serca2a (Ca^2+^-ATPase) activity was measured as the decrease in the absorbance at 340 nm using heart tissue from the various genotypes. Specifically, Serca2a activity was measured by the activity difference between 1 mM CaCl2 with and without the Serca-specific inhibitor TBQ. (O) Quantification of the Serca2a activity in heart tissue (*n* = 3). The data are presented as mean ± SD. (P) Oxidative modifications involving 3-nitrotyrosine and cysteine sulfonation of Serca2a were assessed by IP of the Serca2a protein from heart tissue samples followed by western blot analysis using antibodies against 3-nitrotyrosine, cysteine sulfonate, and Serca2a. (Q) Quantification of oxidative modifications to Serca2a by 3-nitrotyrosine and cysteine sulfonation (*n* = 4). The data are presented as mean ± SD and are analyzed using Student *t* test. *p < 0.05; **p < 0.005. Values for each data point can be found in S1 Data. 3M, 3 months old; 26M, 26 months old; Cc, crude cytosolic fractions; CCCP, carbonyl cyanide m-chlorophenyl hydrazone; Cisd2, CDGSH iron-sulfur domain-containing protein 2; Cp, pure cytosolic fractions; DCF, 2′, 7′-dichlorofluorescein; FCCP, carbonyl cyanide p-trifluoromethoxyphenylhydrazone; Gapdh, glyceraldehyde 3-phosphate dehydrogenase; H, homogenates; IP, immunoprecipitation; KO, knockout; Mc, crude mitochondrial fraction; OCR, oxygen consumption rate; R/A, rotenone/antimycin A; RNS, reactive nitrogen species; ROS, reactive oxygen species; Serca, sarco/endoplasmic reticulum Ca^2+^-ATPase; SR, sarcoplasmic reticulum; Tag, thapsigargin; TBQ, 2,5-Di-tert-butylhydroquinone; TG, transgenic; WT, wild type; YFP, yellow fluorescent protein.

Our previous studies have demonstrated that Cisd2 regulates intracellular Ca^2+^ homeostasis in the liver by modulating Serca2b activity [23]. To gain insights into the molecular mechanism by which Cisd2 deficiency results in cardiac dysfunction, we isolated adult cardiomyocytes [24] from 3M WT, Cisd2KO, and Cisd2TG mice in order to investigate whether Cisd2 deficiency impairs Ca^2+^ homeostasis in these adult cells. Our results reveal that the amplitude of the Ca^2+^ wave during spontaneous beating is significantly decreased in Cisd2KO cardiomyocytes (Fig 4A and 4B; S1–S3 Videos). In addition, the basal cytosolic Ca^2+^ level is significantly elevated in these Cisd2KO cardiomyocytes (Fig 4C). The SR Ca^2+^ store was measured using thapsigargin (Tag) treatment to inhibit Serca2a activity. Notably, the Tag-induced cytosolic Ca^2+^ elevation is significantly reduced in Cisd2KO cardiomyocytes (Fig 4D), indicating that Cisd2 deficiency leads to a reduced Ca^2+^ store in the SR and that Serca2a activity may be diminished thereby, which will affect the reuptake of Ca^2+^ from the cytosol to the SR; these results are similar to those observed in our previous study of hepatocytes [23]. There are two important Ca^2+^ buffering systems in cardiomyocytes, and the other buffering system involves the mitochondria [25]. Mitochondrial Ca^2+^ levels were measured using carbonyl cyanide m-chloro-phenylhydrazone (CCCP) treatment to inhibit mitochondrial Ca^2+^ accumulation. Remarkably, the peak Ca^2+^ released from mitochondria was significantly increased in Cisd2KO cardiomyocytes (Fig 4E). This disruption of cytosolic Ca^2+^ homeostasis appears to diminish contractility during the spontaneous beating of cardiomyocytes that have been isolated from the Cisd2KO mice (Fig 4F and 4G; S4–S6 Videos).

Accumulation of Ca^2+^ in mitochondria occurs through a low-affinity electrogenic mechanism mediated by the mitochondrial Ca^2+^ uniporter [26]. The elevation of cytosolic Ca^2+^ caused by Cisd2 deficiency may have activated this mitochondrial uniporter and led to an increase in mitochondrial Ca^2+^ content. As a consequence, the subsequent mitochondrial Ca^2+^ overload appears to have increased oxidative stress, specifically ROS and reactive nitrogen species (RNS) levels, in cardiac muscle (Fig 4H); this damages mitochondrial functionality, which is evidenced by a reduction in their oxygen consumption rate (OCR) during oxidative phosphorylation (Fig 4I) and a significant decrease in the mitochondrial membrane potential, which was monitored by JC-1 staining of the cardiomyocytes (Fig 4J and 4K). Intriguingly, adult cardiomyocytes isolated from 3M Cisd2TG mice show better performance during mitochondrial functional testing, namely by OCR (Fig 4I) and by JC-1 staining (Fig 4J and 4K). Together, our findings reveal that Cisd2 deficiency disrupts Ca^2+^ homeostasis, which causes mitochondrial defects, and this then compromises the spontaneous contractility of Cisd2KO cardiomyocytes.

Subcellular fractionation of cardiac muscle shows that Cisd2 is enriched in the SR fraction and that Serca2a and Cisd2 are colocalized in the SR fraction (Fig 4L). To visualize whether Cisd2 interacts directly with Serca2a in cells, we performed bimolecular fluorescence complementation assays using split–yellow fluorescent protein (YFP) constructs [27]. The split-YFP assay gave a positive fluorescent signal indicating a direct interaction between Cisd2 and Serca2a (Fig 4M). Next, we examine whether Cisd2 deficiency influences the Serca2a activity. Our result reveals that the activity of Serca2a was significantly decreased by 32% in the myocardium of Cisd2KO mice compared with WT controls (Fig 4N and 4O). However, no significant difference in the protein levels of Serca2a was found (S5A and S5B Fig). Previous studies have revealed that oxidative posttranslational modifications of Serca, including 3-nitrotyrosine and cysteine sulfonation, are able to inhibit Serca activity [28,29]. Indeed, the amounts of oxidative modifications of Serca2a are significantly increased in Cisd2KO cardiac muscle (Fig 4P and 4Q). Interestingly, the activity of Serca2a (Fig 4N and 4O) and the oxidative modifications of Serca2a (Fig 4P and 4Q) in the myocardium of 26M WT mice are similar to those found in 3M Cisd2KO mice. These findings support the hypothesis that Cisd2 is required to regulate Serca2a activity via modulation of the redox status of Serca2a protein, thereby controlling Ca^2+^ homeostasis in cardiomyocytes.

### A persistently high level of Cisd2 delays cardiac aging and attenuates age-related structural and functional decline

**Fig 5:**
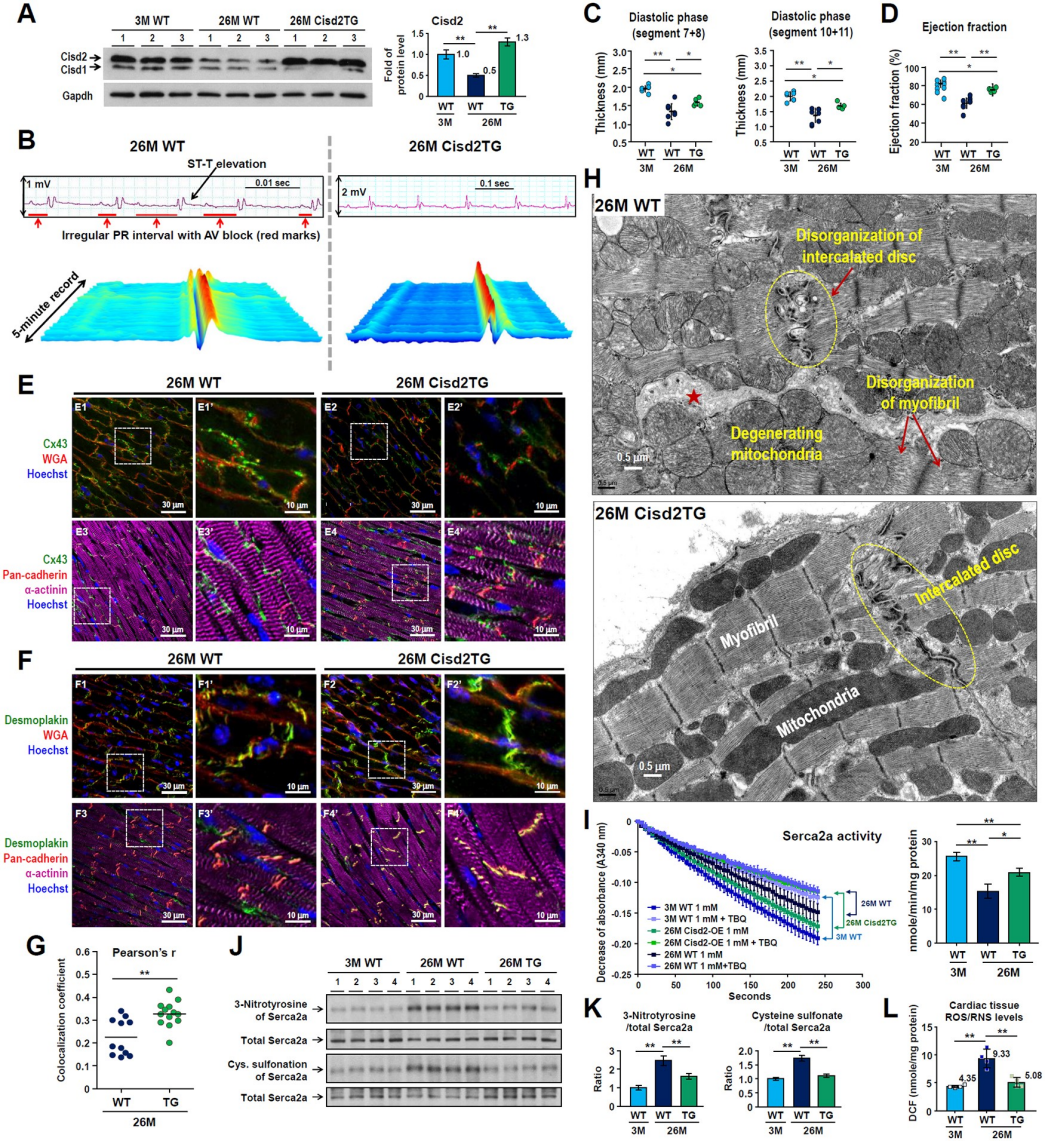
A high level of Cisd2 delays cardiac aging and attenuates age-related structural defects and functional decline during old age. (A) Western blot analysis revealed that Cisd2 protein is about 2.6-fold higher in the cardiac muscle of 26M Cisd2TG mice compared with 26M WT mice. (B) Representative waterfall plots and ECG tracings recorded following anesthesia of mice. Abnormal ECG, namely ST-T elevation and irregular PR interval, were recorded from 26M WT mice. However, relatively normal sinus rhythm of the ECG was recorded from the 26M Cisd2TG mice. (C and D) Analysis of the MRI of diastolic segment 7+8 (anterior LV free wall, C) and diastolic segment 10+11 (interventricular LV wall, D). Data are presented as mean ± SD. Mouse number *n* = 5–6 for each group. (E and F) A high level of Cisd2 alleviates age-related damage to the intercalated discs. Lateralization of gap junctions revealed by Cx43 staining (E) and maldistribution of desmosomes revealed by desmoplakin staining (F) were easily found in the 26M WT mice. However, these defects are barely detectable in the 26M Cisd2TG mice. Method of IF imaging is the same as in Fig 3A and 3E. (G) Colocalization coefficient of gap junctions (Cx43) and intercalated discs (pan-cadherin) was analyzed using Pearson’s correlation. Method is the same as in Fig 3B. (H) A high level of Cisd2 preserves the integrity of cardiac ultrastructure in the Cisd2TG mice at old age. TEM analysis revealed ultrastructural defects in the cardiac muscle of aged WT mice at 26M. The age-related defects include disorganization of intercalated disc; expanded intercellular space (★), which might be caused by breakdown of the intercalated disc; mitochondrial degeneration (e.g., mitochondria with discontinuous or ruptured outer and inner membranes, swollen mitochondria with fewer crista); and disorganization of myofibrils with partial disruption of Z bands and fewer myofilaments. In the Cisd2TG mice, the integrity of mitochondria and intercalated disc, as well as the alignment of myofibrils, is well preserved in the cardiac muscle. (I) Serca2a activity was measured using an enzyme-coupled spectrophotometric assay (*n* = 3–4). Method is the same as in Fig 4N. (J and K) Western blot analysis (J) and quantification (K) of oxidative modifications to Serca2a by 3-nitrotyrosine and cysteine sulfonation using heart tissues (*n* = 4). (L) Quantification of ROS and RNS levels in heart by measuring DCF levels using cardiac tissues (*n* = 4 for each group). The data are presented as mean ± SD and are analyzed by Student *t* test. *p < 0.05; **p < 0.005. Values for each data point can be found in S1 Data. 3M, 3 months old; 26M, 26 months old; AV, atrioventricular; Cisd2, CDGSH iron-sulfur domain-containing protein 2; Cx43, Connexin 43; DCF, 2′, 7′-dichlorofluorescein; ECG, electrocardiography; Gapdh, glyceraldehyde 3-phosphate dehydrogenase; IF, immunofluorescence; LV, left ventricle; MRI, magnetic resonance image; RNS, reactive nitrogen species; ROS, reactive oxygen species; Serca2a, sarco/endoplasmic reticulum Ca^2+^-ATPase; TBQ, 2,5-Di-tert-butylhydroquinone; TEM, transmission electron microscopy; TG, transgenic; WGA, wheat germ agglutinin; WT, wild type.

Since Cisd2 levels decrease during cardiac aging (Fig 1A), we investigate whether maintaining the Cisd2 protein at a persistently high level, comparable to the Cisd2 level at a young age, is able to delay cardiac aging and attenuate the age-related functional decline. We investigated a Cisd2TG mouse model carrying two additional copies of Cisd2 gene (line A302) [23]. During old age, namely 26M, the Cisd2 level of the Cisd2TG mice is about 2.6-fold higher than that of their WT littermates (Fig 5A). Interestingly, age-related cardiac fibrosis was reduced in the 26M Cisd2TG mice (S6A Fig). Quantification revealed no difference in the QTc interval and Tpeak–Tend interval (S6B and S6C Fig) compared to their younger counterparts. Intriguingly, the waterfall plot of 5-minute ECG tracings show a relatively normal pattern of sinus rhythm in the 26M Cisd2TG mice. This contrasted with the presence of an abnormal ECG, namely ST-T elevation and irregular PR interval, in the 26M WT mice (Fig 5B). Cardiac MRI analysis reveals that the hearts of Cisd2TG mice have a better overall performance. The thicknesses of the interventricular septum and left ventricular free wall in the 26M WT mice are significantly thinner than that in the 26M Cisd2TG mice (Fig 5C). Additionally, the ejection fraction of the LV was significantly higher in 26M Cisd2TG mice (Fig 5D). Notably, in 26M WT mice, overt age-related defects affecting the intercalated discs, including lateralization of the gap junctions and maldistribution of desmosomes, are easily detected (Fig 5E and 5F; S6D and S6E Fig); these defects are very similar to those observed in the 3M Cisd2KO mice (Fig 3A and 3E). Importantly, a higher Cisd2 level appears to reduce these structural defects (Fig 5E and 5F) and increase the colocalization coefficient of gap junction protein Cx43 and intercalated disc protein pan-cadherin in the 26M Cisd2TG mice (Fig 5G). TEM examination further reveals that the structural integrity of intercalated disc, sarcomeres, and mitochondria is well preserved in the 26M Cisd2TG mice (Fig 5H). This contrasts with the presence of age-related structural damages in the cardiac muscle of 26M WT mice, including disorganized and partially broken-down intercalated discs and myofibrils, together with swollen and degenerated mitochondria (Fig 5H). Moreover, the age-related decline in Serca2a activity is significantly attenuated (Fig 5I); this is paralleled by a reduction in the level of oxidative modifications of Serca2a (Fig 5J and 5K) and a reduction in the ROS/RNS levels of the cardiac muscle of 26M Cisd2TG mice (Fig 5L).

### Cardiac transcriptomics reveals that similar patterns are present in naturally and prematurely aged mice and that a higher level of Cisd2 during old age results in a younger cardiac transcriptome pattern

To gain insights into the biological relevance of Cisd2 to cardiac aging and to investigate their association with the pathological alterations, we perform RNA sequencing analysis from the following three groups of mice: (1) naturally aged (26M WT versus 3M WT); (2) prematurely aged (3M Cisd2KO versus 3M WT); and (3) long-lived (26M Cisd2TG versus 26M WT). The results are analyzed to identify the presence of differentially expressed genes (DEGs); these are defined as genes with a fold change of >1.5 (either up-regulated or down-regulated) and a significance of *p* < 0.05 compared to their control group.

**Fig 6:**
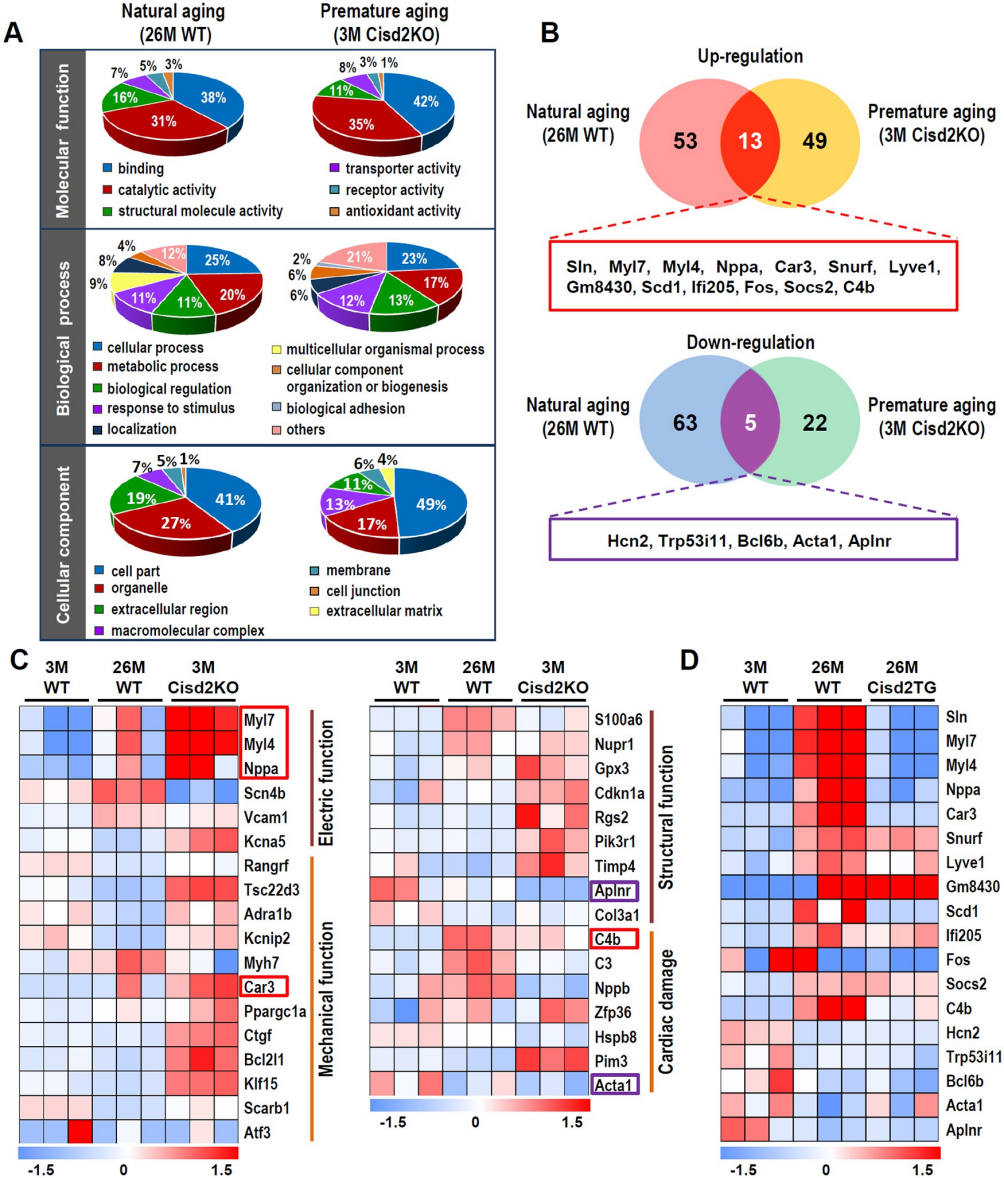
RNA sequencing analyses to examine the DEGs for the naturally and prematurely aged mice as well as the long-lived Cisd2TG mice. (A) A pie chart according to their biological processes, molecular functions, and subcellular localization based on Gene Ontology annotation. (B) A Venn diagram illustrating the common and unique DEGs in the cardiac muscle of natural aging (26M WT versus 3M WT) and premature aging (3M Cisd2KO versus 3M WT). Numbers represent the amount of significantly changed genes (fold change > 1.5, *p* < 0.05) in each pairwise comparison and in their respective overlaps. Complete gene lists are provided in S7 Fig. (C) Classification of cardiac functional pathways by IPA illustrates the significant canonical pathways related to cardiac electric and mechanical functions, as well as cardiac structure and damage. Each column represents an animal sampled at depicted age. Hue represents the Z-score (RPKM minus mean over SD) of each gene (row) of 3M WT, 26M WT, and 3M Cisd2KO mice. The genes highlighted in red boxes (up-regulation) and purple boxes (down-regulation) are genes present in the common DEGs of natural aging and premature aging mice as shown in Fig 6B, respectively. (D) Comparison of the heatmaps of the common DEGs (up- and down-regulation), which were identified by comparing the DEGs of naturally and prematurely aged mice, for the young mice (3M WT), naturally aged mice (26M WT), and long-lived Cisd2TG mice (26M Cisd2TG). Values for each data point can be found in S1 Data. 3M, 3 months old; 26M, 26 months old; Cisd2KO, CDGSH iron-sulfur domain-containing protein 2 knockout; Cisd2TG, CDGSH iron-sulfur domain-containing protein 2 transgenic; DEG, differentially expressed gene; RPKM, reads per kilobase million; IPA, Ingenuity Pathway Analysis; Sln, sarcolipin; WT, wild type.

Gene Ontology classification reveals that most DEGs in two datasets (26M WT versus 3M WT; 3M Cisd2KO versus 3M WT) are involved in similar process, namely molecular functions, biological processes, and/or cellular components (Fig 6A). This indicates the presence of commonly altered functional pathways in the cardiac muscles of naturally and prematurely aged mice. Among the up-regulated DEGs, 66 and 62 genes were recognized in the naturally and prematurely aged mice, respectively; there were 13 common genes observed in these two DEG datasets (sarcolipin [Sln], Myl7, Myl4, Nppa, Car3, Snurf, Lyve1, Gm8430, Scd1, Ifi205, Fos, Socs2, C4b) (Fig 6B; S7A and S7B Fig). Among the down-regulated DEGs, 68 and 27 genes were recognized in the naturally and prematurely aged mice, respectively; there were five common genes observed in these two DEG datasets (Hcn2, Trp53i11, Bcl6b, Acta1, Aplnr) (Fig 6B; S7A and S7B Fig). The DEGs were further annotated by Ingenuity Pathway Analysis to determine the implications of these expression changes regarding cardiac function and structure. Among the statistically significant canonical pathways, many of the DEGs were found to be involved in electrical and mechanical functions, as well as in cardiac structure and in damage (Fig 6C).

To study the effect of a high level of Cisd2 on cardiac gene expression, we compare the heatmap patterns of the common DEGs (up-regulated and down-regulated; S2 Table) obtained from the young mice (3M WT), the naturally aged mice (26M WT), and the long-lived Cisd2TG mice at 26M. Strikingly, the pattern of heatmap analysis of the 26M Cisd2TG mice is closest to and most like that of the 3M WT mice (Fig 6D), which indicates that a higher level of Cisd2 appears to delay cardiac aging and bring about a younger pattern of cardiac gene expression. Moreover, the mRNA levels of genes related to cardiac necrosis and cell death pathways are up-regulated in the 3M Cisd2KO and 26M WT mice; furthermore, the heatmap pattern of the 26M Cisd2TG is very similar to that of the 3M WT mice (S7C Fig). The presence of cardiac cell death was further confirmed by an increased level of cleaved Caspase-3 in the Cisd2KO mice (S7D Fig), and this is consistent with the observation that a smaller heart is present in Cisd2KO mice (Fig 1B and 1C). When we examined the pathways related to ROS metabolic regulation and the responses to ROS, the heatmap pattern of 3M Cisd2KO is most similar to that of the 26M WT mice (S8A Fig), whereas the 26M Cisd2TG is most similar to that of the 3M WT mice (S8B Fig).

## Discussion

**Fig 7:**
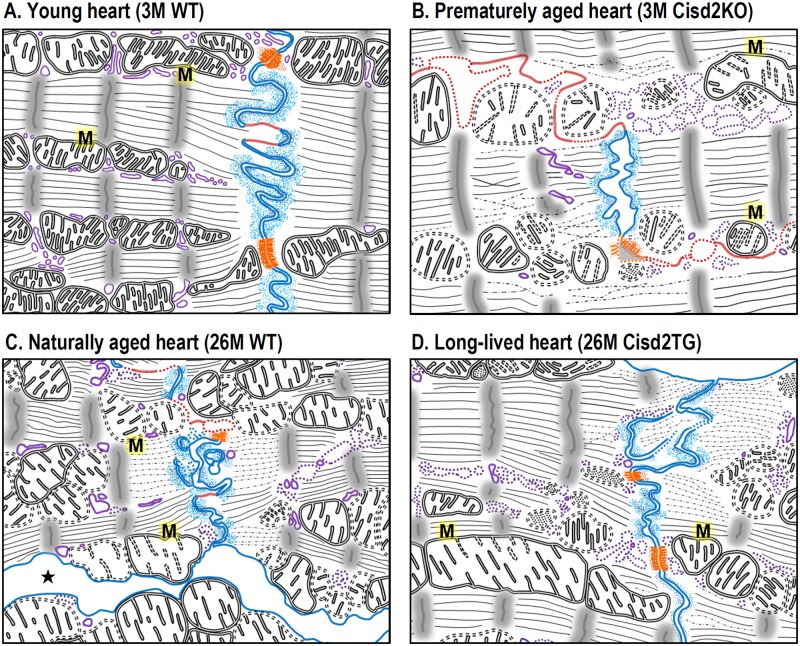
Cisd2 plays an essential role to maintain the integrity of intercalated disc and ultrastructure of cardiomyocytes during cardiac aging. (A) In the young heart of 3M WT mice, the three types of cell junction that make up the intercalated disc—namely, gap junctions (marked in red), desmosomes (marked in orange), and fascia adherens (marked in blue)—can be easily identified. In addition, their SR (marked in purple), mitochondria (“M”), and myofibrils appear morphologically intact. (B) In the prematurely aged heart of 3M Cisd2KO mice, their fascia adherens are shortened and broken down; gap junctions are extended and fragmented; desmosomes are degenerated. Additionally, SR, mitochondria, and myofibrils are partially degenerated. (C) In the naturally aged heart of 26M WT mice, their fascia adherens are disorganized and partially broken down; gap junctions are extended and fragmented; desmosomes are partially degenerated. Furthermore, expanded intercellular space (★) caused by degeneration of intercalated disc was found. SR, mitochondria, and myofibrils are partially degenerated and disorganized. (D) In the long-lived heart of 26M Cisd2TG mice, the integrity of intercalated disc and ultrastructure of organelles and myofibrils are much better preserved in the cardiac muscle. 3M, 3 months old; 26M, 26 months old; Cisd2, CDGSH iron-sulfur domain-containing protein 2; KO, knockout; SR, sarcoplasmic reticulum; TG, transgenic; WT, wild type.

Here, we provide evidence for the first time to indicate that Cisd2 plays an essential role in maintaining normal cardiac function. Without Cisd2, a young mouse has an old heart with deteriorated cardiac functioning. Conversely, by maintaining a persistently high level of Cisd2 throughout the mouse’s lifetime, an old mouse has a younger and healthier heart. Five novel findings are pinpointed. Firstly, Cisd2 is pivotal to successfully maintain cardiac functioning and a normal cardiac structure. Cisd2 deficiency in Cisd2KO mice impairs electromechanical performance and causes myocardial degeneration at a young age. Secondly, the level of Cisd2 protein present in the heart is crucial to intercalated disc integrity, which allows individual cardiomyocytes to synchronously act as a single functional organ. In Cisd2KO mice, Cisd2 deficiency results in intercalated disc defects, namely lateralization of gap junctions, the maldistribution of desmosomes, and a breakdown of the fascia adherens. This apparently leads to electromechanical impairment. However, in Cisd2TG mice, persistently high Cisd2 levels appear to preserve the intercalated discs through into old age; this is in contrast to the presence of significant age-related damage affecting the intercalated discs of naturally aged WT mice. The essential role of Cisd2 in maintaining the integrity of the intercalated discs is summarized in Fig 7. Thirdly, Cisd2 interacts directly with Serca2a and mediates Serca2a activity via modulation of the protein’s posttranslational oxidative modifications, which in turn regulates SR Ca^2+^ reuptake and maintains intracellular Ca^2+^ homeostasis. Cisd2 deficiency disrupts Ca^2+^ homeostasis, increasing basal cytosolic Ca^2+^ levels, which causes mitochondrial Ca^2+^ overload and compromises mitochondrial functioning. Fourthly, Cisd2 is essential to maintaining the integrity of mitochondrial ultrastructure and the normal functioning of oxidative phosphorylation. In addition to the SR, Cisd2 is also located in the MOM [10]. Our previous reports [10,11] and the current study have revealed that Cisd2 appears to preserve mitochondrial respiratory function and membrane potentials, as well as minimize ROS production; consequently, this will reduce intracellular oxidative stress and Serca2a oxidative modifications, thereby preserving normal ultrastructure and function of cardiomyocytes. Finally, the level of Cisd2 present in the cardiac muscle of naturally aged mice is significantly reduced to approximately 50% of that found in young mice. Intriguingly, maintaining a persistently high level of Cisd2 delays cardiac aging, attenuates the development of age-related structural defects, and prevents functional decline. Importantly, this persistent level of Cisd2 throughout life results in a cardiac transcriptome that is similar to the pattern found in young mice and is distinctly different to that found in naturally aged mice.

### Cisd2 preserves Serca2a activity during cardiac aging

Despite the successful rescue of cardiac performance and survival via restoration of Serca2a by gene transfer in animals [30,31], the Phase II human trial has failed [32]. The activity of Serca family proteins is inhibited by posttranslational oxidative modifications involving 3-nitrotyrosine and cysteine sulfonation [28,29], which are mediated by ROS. In the aged heart, increased oxidative stress is well recognized, and this oxidative stress has been implicated in the pathophysiology of cardiac aging [33]. Accordingly, protecting Serca2a protein from posttranslational oxidative modifications, rather than increasing the amount of Serca2a protein present, is an alternative strategy to increasing Serca2a activity. Indeed, in Cisd2TG mice, the overexpressed Cisd2 appears to decrease ROS and protect the Serca2a from oxidative modifications during cardiac aging, thereby attenuating the cardiac functional decline associated with old age. This may provide a direct beneficial effect on cardiac electromechanical performance via maintaining intracellular Ca^2+^ homeostasis. There are three possible explanations as to how Cisd2 modulates the redox status of Serca2a in cardiomyocytes. Firstly, Cisd2 protein contains a CDGSH domain, which binds a redox-active 2Fe-2S cluster. This is oriented toward the cytosol, and it seems quite likely that Cisd2 directly interacts with Serca2a and maintains Serca2a in a reduced state via the redox capacity of this CDGSH domain. Secondly, Cisd2 may reduce the availability of tyrosines and cysteines on Serca2a via protein–protein interaction and thus protect these amino acids, which are the targets of oxidative modification. Finally, the redox active of the CDGSH domain of Cisd2 may be involved in regulating the general redox status of the cell, thereby reducing overall intracellular ROS levels, which will indirectly help to protect Serca2a activity. Further mechanistic investigations will be required in order to obtain a better understanding of how Cisd2 modulates Serca2a activity in cardiomyocytes.

Interestingly, our results from a transcriptomics analysis using RNA sequencing revealed that Sln, an important regulator of Serca2a activity, is up-regulated in the hearts of both naturally aged (26M WT) and prematurely aged (3M Cisd2KO) mice (Fig 6B; S9 Fig). In contrast, the expression level of Sln is down-regulated in the hearts of long-lived (26M Cisd2TG) mice (Fig 6D; S9 Fig). Since overexpression of Sln is known to result in a reduction in the Ca^2+^ affinity of Serca2a and in Ca^2+^ transient amplitude [34], these results suggest that there might be an interplay between the three crucial protein factors, namely Serca2a, Sln, and Cisd2. These together might coordinate the regulation of Ca^2+^ homeostasis in a multifactorial Cisd2-dependent process during cardiac aging. The mechanism is currently unknown but is worth future investigation.

### Disrupted Ca^2+^ homeostasis in aging hearts

Cytosolic Ca^2+^ homeostasis in cardiomyocytes is finely tuned by multiple regulatory processes that involve a number of regulators. The L-type Ca^2+^ channels (LTCCs) and ryanodine receptors (RyR2s) are able to increase the level of intracellular Ca^2+^, whereas Na^+^/Ca^2+^ exchanger (NCX1, the major cardiac isoform) and Serca2a are able to decrease the level of cytosolic Ca^2+^. Previous studies have revealed that cytosolic Ca^2+^ overload in an aging heart can result from (1) RyR2 glycation–induced calcium leakage and mitochondrial damage [35], (2) a reduction in Serca2a-mediated Ca^2+^ reuptake into the SR [36], (3) delayed inactivation and a reduced LTCC density at the T-tubules [37], and (4) an increase in forward NCX1 activity in the senescent myocardium [38]. Furthermore, a previous study has shown that, in the aged cardiomyocytes, the abnormal increase in spark activity may be attributable to an increase in the opening frequency of RyR2, which undergoes posttranslational modification during aging [36]. During cardiac aging, the altered phosphorylation and glycation status of RyR2 and the changes in the activity of various associated kinases—including protein kinase A, Ca^2+^/calmodulin-dependent kinase II, phosphatases, and glyoxalase-1—may all contribute to SR calcium leakage and the increase in cytosolic Ca^2+^ level; eventually these will lead to mitochondrial damage [36].

In this study, we provide evidence demonstrating that Serca2a also plays a crucial role in maintaining Ca^2+^ homeostasis during cardiac aging. A decrease in Serca2a activity, whether caused by natural aging or by Cisd2KO, prolongs Ca^2+^ reuptake into the SR after cardiomyocytic excitation. This in turn leads to an elevated cytosolic Ca^2+^ level and thus diastolic dysfunction. Intriguingly, a persistently high level of Cisd2 appears to preserve Serca2a function and helps to maintain Ca^2+^ homeostasis during cardiac aging. However, it is possible that there may also be concurrent age-dependent alterations in multiple other key regulators that are involved in modulating intracellular Ca^2+^ homeostasis, such as concurrent changes in Serca2a, RyR2, LTCC, and NCX1. Accordingly, it would be of great interest in the future to simultaneously study the effect of Cisd2 on these key regulators during cardiac aging.

### An elevation of cytosolic Ca^2+^ may cause intercalated disc defects during aging

The age-related disorganization of intercalated disc has been reported previously in the aged mouse heart [39]. The disorganization and breakdown of the intercalated discs in prematurely aged Cisd2KO and in naturally aged 26M WT mice impedes the proper propagation of electrical impulses throughout the myocardium, which is conducted by the gap junctions. Furthermore, there is damage to the mechanical joints between adjacent cardiomyocytes. These joints allow the cardiomyocytes to work as a single functional unit, and these connections involve the desmosomes. The elevated cytosolic Ca^2+^, caused by Cisd2 deficiency, seems to disrupt the protein organization of the intercalated disc. In terms of the desmosome, its component protein, desmoplakin, is required for intermediate filament anchorage to the adherent sites of the desmosome. The association between desmoplakin and intermediate filaments, as well as desmosome assembly, is regulated by protein kinase Cα (PKCα) [40]. Normally, regulation of PKCα involves its interaction with the cell membrane where the protein presence involves Ca^2+^. In the absence of Serca2, the membrane translocation of both PKCα and desmoplakin was significantly impaired in epidermal-derived cell lines, suggesting that a deficiency in Serca2 will disrupt desmosome assembly [41]. In the cardiomyocytes, the PKCα-dependent pathway of desmosome assembly has been identified [42]. Importantly, Lange and colleagues showed that during the end stage of dilated cardiomyopathy, PKCα signaling is elevated and concentrated within the intercalated discs of cardiomyocytes; in this way, chronic uninhibited PKCα activity leads to heart failure [43].

When gap junctions are explored, Cx43 has been found to be the major component protein that forms gap junction in hearts. The Cx43 protein has a short half-life of 1–3 hours [44]; accordingly, there needs to be high efficiency associated with this protein’s dynamic turnover; this will involve constant disassembly, assembly, and remodeling. In diseased hearts, a high level of cytosolic Ca^2+^ will activate calmodulin/calcium-calmodulin protein kinase II (CaM/CaMKII), which is involved in cardiac arrhythmias [45]. The activated CaM/CaMKII directly interacts with Cx43 and phosphorylates Cx43 protein, leading to Cx43 remodeling, including lateralization, as well as impairment of electric propagation via gap junctions [22]. Interestingly, genetic inhibition of CaMKII improves cardiac conduction and enhances the localization of Cx43 to the intercalated disc [46]. Furthermore, Ca^2+^-saturated CaM can directly bind to and activate calcineurin A (CnA), which is the catalytic subunit of calcineurin, a serine-threonine phosphatase [45]. Overexpression of a constitutively active form of CnA in mice results in an impairment of Cx43 phosphorylation and a reduction in Cx43 protein levels [47]. Together, these studies suggest that an elevated cytosolic Ca^2+^ in the heart activates CaM/CaMKII and CaM/CnA signaling, which in turn disturbs the assembly and remodeling of gap junctions via phosphorylation modulation and subcellular localization of Cx43.

### Clinical implications: CISD2 is a drug target for treating age-related CVD

Research on cardiac aging is crucial to the development of effective diagnostic and therapeutic strategies that will prevent and treat age-related CVDs, which are the number-one cause of death globally in the elderly [2]. Development of therapeutic agents that bring about an effective enhancement of Cisd2 expression is one potential therapeutic strategy. The up-regulation of Cisd2 expression by a Cisd2 activator should help to protect the integrity of intercalated discs, enhance mitochondria and Serca2a activity, and maintain intracellular Ca^2+^ homeostasis, thereby attenuating age-related cardiac dysfunction and preventing a subsequent progression to heart failure.

Furthermore, using another rodent model, an age-related decline in the Ca^2+^-sequestering activity of Serca2a has been reported in the myocardium [48]. In humans, the integrity of intercalated discs undergoes remodeling with age, and this brings about cardiac dysfunction in older patients [49]. Furthermore, large-scale ECG analysis has identified several age-related electrical features associated with cardiac aging [50,51], and most of these features are recapitulated with accelerated speed in Cisd2KO mice. Conversely, many features of cardiac aging are delayed in Cisd2TG mice. Thus, the Cisd2KO and Cisd2TG mice are able to serve as an excellent animal platform for studying cardiac aging and as a test platform for intervention strategy developed in the future.

## Methods

### Ethics statement

The animal protocols followed local animal ethics regulations and were approved by the Institutional Animal Care and Use Committee of Chang Gung Memorial Hospital (approval no. 2014112301) and National Yang-Ming University (approval no. 1021218).

### Mouse models

Cisd2KO [10] and Cisd2TG [23] mice were generated as previously described. Male mice were used for all experiments. All mouse lines have a pure or congenic C57BL/6 background and were bred/housed in a specific pathogen–free facility.

### Statistical analysis

The data are presented as mean ± SD or mean ± SEM, as described in the figure legends. Comparisons between the two groups were done using a two-tailed Student *t* test. When analyzing statistical differences among groups, *p* < 0.05 was considered significant. Statistical analysis was made by using the software Graphpad Prism 6.0.

Expanded methods are provided in the supporting information (S1 Text).

## Supporting information

S1 Data Underlying data for main figures and supporting figures. Excel spreadsheet containing, in separate sheets, the underlying numerical data and statistical analysis for Figs 1A, 1C, 1F, 1G, 2E–2I, 3B–3G, 4A–4I, 4K, 4N, 4O, 4Q, 5A, 5C, 5D, 5G, 5I, 5K, 5L and 6, as well as S1A, S1B, S2A–S2F, S3E, S3F, S5B, S6B, S6C, S6E, S7A–S7D, S8A, S8B and S9 Figs.
(XLSX)

S1 Table Electrocardiographic characteristics of WT, Cisd2KO, and Cisd2TG mice at different ages. Related to Fig 2. Cisd2KO, CDGSH iron-sulfur domain-containing protein 2 knockout; Cisd2TG, CDGSH iron-sulfur domain-containing protein 2 transgenic; WT, wild type.
(DOCX)

S2 Table Common DEGs (up- and down-regulation) of the naturally aged WT mice at 26M and prematurely aged Cisd2KO mice at 3M. Related to Fig 6. 3M, 3 months old; 26M, 26 months old; Cisd2KO, CDGSH iron-sulfur domain-containing protein 2 knockout; DEG, differentially expressed gene; WT, wild type.
(DOCX)

S1 Fig Myocardial mass/body weight (A), body weight (B), Sirius Red/Fast Green staining of collagen for the papillary muscle (C), heart rate/minute (D), noninvasive tail-cuffs measurement of systolic blood pressure (E), diastolic blood pressure (F). **Related to** Figs 1 and 2. The heart weight (A) and body weight (B) were measured at the time of euthanasia. (C) High-power section of the papillary muscle with Sirius Red/Fast Green staining of Fig 1D. An increase in the collagen fibers present is notable in the old WT mice at 26M and in the Cisd2KO mice at 3M and 6M. Scale bar, 50 μm. Heart rate (D) was measured from 5 minutes of sequential beats of whole ECG tracings. Systolic blood pressure (E) and diastolic blood pressure (F) of WT, Cisd2KO, and Cisd2TG were measured from conscious mice at designated ages using the noninvasive tail-cuffs BP-2000 Blood Pressure Analysis System (Visitech Systems, Apex, NC, USA). Blood pressure values were recorded 20 times in rapid succession, and the mean value was generated for each individual mouse. The data are presented as mean ± SD. *p < 0.05; **p < 0.005. Values for each data point can be found in S1 Data. 3M, 3 months old; 6M, 6 months old; 26M, 26 months old; Cisd2KO, CDGSH iron-sulfur domain-containing protein 2 knockout; Cisd2TG, CDGSH iron-sulfur domain-containing protein 2 transgenic; ECG, electrocardiography; WT, wild type.
(TIF)

S2 Fig Measurements of thyroid hormones; serum markers related to renal function, blood glucose, and insulin levels; serum markers related to lipid metabolism; and serum electrolytes for the WT and Cisd2KO mice at 3 months old. **Related to** Fig 2. (A) Serum T_3_ and T_4_ levels (mouse number *n* = 3). (B) Serum BUN and creatinine levels (mouse number *n* = 3). (C) Blood glucose levels after 2 and 12 hours of fasting (mouse number *n* = 4–8). (D) Serum insulin levels after 12 hours of fasting (mouse number *n* = 3–5). (E) Serum TCHO and TriG levels (mouse number *n* = 7–8). (F) Serum Ca^2+^, Mg^2+^, Na^+^, K^+^, and Cl^−^ levels (mouse number *n* = 3). The data are presented as mean ± SD. *p < 0.05; **p < 0.005. Values for each data point can be found in S1 Data. BUN, blood urea nitrogen; Cisd2KO, CDGSH iron-sulfur domain-containing protein 2 knockout; T_3_, triiodothyronine; T_4_, thyroxine; TCHO, total cholesterol; TriG, triacylglycerol; WT, wild type.
(TIF)

S3 Fig Protein levels of the intercalated disc–related proteins in the cardiac muscle of WT and Cisd2KO mice. **Related to** Fig 3. Western blot analyses of Cx43 (A), phosphorylated form of Cx43 (Ser368) (B), desmoplakin (C), phosphorylated form of desmoplakin (D), vinculin and α-actinin (E), and pan-cadherin (F) for the heart tissues of WT and Cisd2KO mice at 3 months old. There are four animals for each group of mice. The data are presented as mean ± SD. *p < 0.05; **p < 0.005. Values for each data point can be found in S1 Data. Cisd2KO, CDGSH iron-sulfur domain-containing protein 2 knockout; Cx43, Connexin 43; WT, wild type.
(TIF)

S4 Fig Ultrastructural abnormalities in the cardiac muscle of Cisd2KO at 6M. **Related to** Fig 3. The two representative TEM micrographs revealed severe ultrastructure defects in the cardiac muscle of 6M Cisd2KO mice. Notably, SR degeneration with dilated cisternae and myofibril degeneration with a decreased number of myofibrils, as well as partial disruption of some Z bands, were more severe and easily detected at 6 months of age. Moreover, the severity of the mitochondrial damages—including mitochondria with ruptured outer and inner membranes, swollen mitochondria, and pale mitochondria with fewer cristae—is also more obvious in the Cisd2KO heart at 6M. 6M, 6 months old; Cisd2KO, CDGSH iron-sulfur domain-containing protein 2 knockout; SR, sarcoplasmic reticulum; TEM, transmission electron microscopy.
(TIF)

S5 Fig Analysis of Serca2a protein levels in the cardiac muscle. **Related to** Fig 4. (A and B) Western blot analysis (A) and quantification (B) of Serca2a protein levels in the hearts of WT (*n* = 4), Cisd2KO (*n* = 4), and aged WT mice (*n* = 6). The data are presented as mean ± SD. Values for each data point can be found in S1 Data. Cisd2KO, CDGSH iron-sulfur domain-containing protein 2 knockout; Serca2a, sarco/endoplasmic reticulum Ca^2+^-ATPase; WT, wild type.
(TIF)

S6 Fig Pathological and functional examination of 26M WT and 26M Cisd2TG mice. **Related to** Fig 5. (A) Long-axis low-power section of heart and high-power section of papillary muscle stained with Sirius Red/Fast Green for detection of collagen. Representative cardiac MRI is also shown. (B) Corrected QT interval measurements made from 5-minute sequential beats of whole ECG tracings from baseline. (C) Tpeak–Tend interval measurements made from 5 minutes of sequential beats of whole ECG tracings from baseline. (D and E) Western blot analysis (D) and quantification (E) of protein levels of Cx43, desmoplakin, and phosphorylated desmoplakin in the hearts of WT and Cisd2TG mice (*n* = 3). The data are presented as mean ± SD. *p < 0.05. Values for each data point can be found in S1 Data. 26M, 26 months old; Cisd2TG, CDGSH iron-sulfur domain-containing protein 2 transgenic; Cx43, Connexin 43; ECG, electrocardiography; MRI, magnetic resonance imaging; WT, wild type.
(TIF)

S7 Fig RNA sequencing analysis of DEGs and increase of cell death markers in the cardiac muscle of Cisd2KO mice. **Related to** Fig 6. (A) List of 116 differentially expressed mRNAs (up 53 + down 63) uniquely identified in the hearts of naturally aged mice (26M WT versus 3M WT). (B) List of 71 differentially expressed mRNAs (up 49 + down 22) uniquely identified in the hearts of prematurely aged mice (3M Cisd2KO versus 3M WT). (C) Heatmap illustrating the mRNA levels of genes related to cardiac necrosis/cell death pathway by IPA classification. The mRNA expression level was analyzed by RNA sequencing. (D) Western blot analyses and quantification of FL-Caspase-3 and cleaved Caspase-3 in the hearts of WT and Cisd2KO mice at 6 months old (*n* = 4). The data are presented as mean ± SD. *p < 0.05. Values for each data point can be found in S1 Data. 3M, 3 months old; 26M, 26 months old; Cisd2KO, CDGSH iron-sulfur domain-containing protein 2 knockout; DEG, differentially expressed gene; FL, full-length; IPA, Ingenuity Pathway Analysis; WT, wild type.
(TIF)

S8 Fig RNA sequencing analysis of DEGs related to regulation of ROS and response to ROS in the cardiac muscles by IPA classification. **Related to** Fig 6. (A) Heatmap illustrating the DEGs identified in the hearts of naturally aged mice (26M WT) and prematurely aged mice (3M Cisd2KO) compared with young mice (3M WT). (B) Heatmap illustrating the DEGs identified in the hearts of naturally aged mice (26M WT) and long-lived mice (26M Cisd2TG) compared with young mice (3M WT). Values for each data point can be found in S1 Data. 3M, 3 months old; 26M, 26 months old; Cisd2KO, CDGSH iron-sulfur domain-containing protein 2 knockout; Cisd2TG, CDGSH iron-sulfur domain-containing protein 2 transgenic; DEG, differentially expressed gene; IPA, Ingenuity Pathway Analysis; ROS, reactive oxygen species; WT, wild type.
(TIF)

S9 Fig The expression level of Sln mRNA is up-regulated in naturally aged (26-month-old WT) and prematurely aged (3-month-old Cisd2KO) hearts, whereas the expression level of Sln mRNA is significantly down-regulated in the long-lived (26-month-old Cisd2TG) hearts. **Related to** Fig 6 **and discussion**. The Sln mRNA levels were obtained from a transcriptomics analysis using RNA sequencing. The data are presented as mean ± SD. *p < 0.05 by one-tailed *t* test. Values for each data point can be found in S1 Data. Cisd2KO, CDGSH iron-sulfur domain-containing protein 2 knockout; Cisd2TG, CDGSH iron-sulfur domain-containing protein 2 transgenic; RPKM, reads per kilobase of exon model per million reads; Sln, sarcolipin; WT, wild type.
(TIF)

S1 Video Ca^2+^ wave in a representative primary cardiomyocyte obtained from an adult WT mouse at 3 months old. WT, wild type.
(MP4)

S2 Video Ca^2+^ wave in a representative primary cardiomyocyte obtained from an adult Cisd2KO mouse at 3 months old. Cisd2KO, CDGSH iron-sulfur domain-containing protein 2 knockout.
(MP4)

S3 Video Ca^2+^ wave in a representative primary cardiomyocyte obtained from an adult Cisd2TG mouse at 3 months old. Cisd2TG, CDGSH iron-sulfur domain-containing protein 2 transgenic.
(MP4)

S4 Video Spontaneous beating of a representative primary cardiomyocyte obtained from an adult WT mouse at 3 months old. WT, wild type.
(MP4)

S5 Video Spontaneous beating of a representative primary cardiomyocyte obtained from an adult Cisd2KO mouse at 3 months old. Cisd2KO, CDGSH iron-sulfur domain-containing protein 2 knockout.
(MP4)

S6 Video Spontaneous beating of a representative primary cardiomyocyte obtained from an adult Cisd2TG mouse at 3 months old. Cisd2TG, CDGSH iron-sulfur domain-containing protein 2 transgenic.
(MP4)

S1 Text Supporting methods. (DOCX)

## Abbreviations

10M: 10 months old
26M: 26 months old
3M: 3 months old
6M: 6 months old
APC: atrial premature complex
CaM/CaMKII: calmodulin/calcium-calmodulin protein kinase II
Cc: crude cytosolic fractions
CCCP: carbonyl cyanide m-chloro-phenylhydrazone
CISD2: CDGSH iron-sulfur domain-containing protein 2
CnA: calcineurin A
Cp: pure cytosolic fractions
CVD: cardiovascular disease
Cx43: Connexin 43
DCF: 2′, 7′-dichlorofluorescein
DEG: differentially expressed gene
ECG: electrocardiography
ER: endoplasmic reticulum
FCCP: carbonyl cyanide p-trifluoromethoxyphenylhydrazone
Gapdh: glyceraldehyde 3-phosphate dehydrogenase
H: homogenates
IF: immunofluorescence
IP: immunoprecipitation
KO: knockout
LTCC: L-type Ca^2+^ channel
LV: left ventricle
MAM: mitochondria-associated ER membrane
Mc: crude mitochondrial fraction
MOM: mitochondrial outer membrane
MRI: magnetic resonance imaging
NCX1: Na^+^/Ca^2+^ exchanger
OCR: oxygen consumption rate
PKCα: protein kinase Cα
QTc: corrected QT
R/A: rotenone/antimycin A
RNS: reactive nitrogen species
ROS: reactive oxygen species
RPKM: reads per kilobase million
RyR2: ryanodine receptor
Serca2a: sarco/endoplasmic reticulum Ca^2+^-ATPase
Sln: sarcolipin
SR: sarcoplasmic reticulum
Tag: thapsigargin
TBQ: 2,5-Di-tert-butylhydroquinone
TEM: transmission electron microscopy
TG: transgenic
VPC: ventricular premature complex
WFS: Wolfram syndrome
WT: wild type
YFP: yellow fluorescent protein
