# Supplementary material for: Cisd2 is essential to delaying cardiac aging and to maintaining heart functions
Source: PLoS Biol. 2019 Oct 8;17(10):e3000508. doi: 10.1371/journal.pbio.3000508 (PMC6799937; doi:10.1371/journal.pbio.3000508)
Supplement: S1 Table — Related to Fig 2. Cisd2KO, CDGSH iron-sulfur domain-containing protein 2 knockout; Cisd2TG, CDGSH iron-sulfur domain-containing protein 2 transgenic; WT, wild type. (DOCX) [file pbio.3000508.s002.docx]

**S1 Table. Electrocardiographic characteristics of WT, Cisd2KO, and Cisd2TG mice at different ages.**

**Related to Figure 2.**

| **Age** | **3 M** | | **6 M** | | **26 M** | |
| --- | --- | --- | --- | --- | --- | --- |
| **Genotype** | **WT** | **Cisd2KO** | **WT** | **Cisd2KO** | **WT** | **Cisd2TG** |
| **Heart Rate (mimute^-1^)** | **470 ± 55** | **483 ± 53** | **465 ± 24** | **455 ± 48** | **479 ± 66** | **501 ± 49** |
| **PR Interval (ms)** | **45.9 ± 6.1** | **45.0 ± 3.6** | **44.3 ± 3.8** | **43.8 ± 2.6** | **45.6 ± 4.7** | **49.5 ± 4.4** |
| **P Duration (ms)** | **15.8 ± 3.3** | **14.2 ± 1.6** | **17.1 ± 3.1** | **13.4 ± 1.9** | **16.4 ± 5.2** | **18.4 ± 1.7** |
| **QRS Interval (ms)** | **9.2 ± 0.8** | **9.6 ± 0.8** | **9.0 ± 0.7** | **9.9 ± 0.7** | **10.2 ± 1.7** | **9.3 ± 0.5** |
| **QTc (ms)** | **48.2 ± 6.1** | **55.7 ± 4.2*** | **46.2 ± 6.9** | **56.7 ± 5.6*** | **58.3 ± 11.0^‡^** | **49.9 ± 1.2^#^** |
| **ST Height (mV)** | **0.08 ± 0.05** | **0.11 ± 0.03** | **0.06 ± 0.01** | **0.11 ± 0.05** | **0.07 ± 0.03** | **0.06 ± 0.01** |
| **Tpeak Tend Interval (ms)** | **5.2 ± 0.6** | **7.2 ± 1.4*** | **5.9 ± 0.6** | **7.5 ± 1.8*** | **7.7 ± 3.0^‡^** | **6.0 ± 0.8^#^** |

* P<0.05 when compared Cisd2KO to their age-matched WT control.

^#^ P<0.05 when compared 26-month old (26M) Cisd2TG to their age-matched 26M WT control.

^‡^ P<0.05 when compared 26M WT to 6M WT mice.

The data are presented as mean ± SD. Comparisons between two groups were done by Student’s t test.
