# Supplementary material for: Cisd2 is essential to delaying cardiac aging and to maintaining heart functions
Source: PLoS Biol. 2019 Oct 8;17(10):e3000508. doi: 10.1371/journal.pbio.3000508 (PMC6799937; doi:10.1371/journal.pbio.3000508)
Supplement: S2 Table — Related to Fig 6. 3M, 3 months old; 26M, 26 months old; Cisd2KO, CDGSH iron-sulfur domain-containing protein 2 knockout; DEG, differentially expressed gene; WT, wild type. (DOCX) [file pbio.3000508.s003.docx]

**S2 Table. Common DEGs (up- and down-regulation) of the naturally aged WT mice at 26-month old (26M) and prematurely aged Cisd2KO mice at 3-month old (3M).**

**Related to Figure 6.**

| **Gene** | **Description** | **Location** | **Family** |
| --- | --- | --- | --- |
| **Sln*** | **sarcolipin** | **Cytoplasm** | **other** |
| **Myl7*** | **myosin light chain 7** | **Cytoplasm** | **enzyme** |
| **Myl4*** | **myosin light chain 4** | **Cytoplasm** | **other** |
| **Nppa*** | **natriuretic peptide A** | **Extracellular Space** | **other** |
| **Car3*** | **carbonic anhydrase 3** | **Cytoplasm** | **enzyme** |
| **Snurf** | **SNRPN upstream reading frame** | **Nucleus** | **other** |
| **Lyve1** | **lymphatic vessel endothelial hyaluronan receptor 1** | **Plasma Membrane** | **transmembrane receptor** |
| **Gm8430** | **predicted pseudogene 8430** | **Other** | **other** |
| **Scd1** | **stearoyl-CoA desaturase 1** | **Cytoplasm** | **enzyme** |
| **Ifi205** | **interferon activated gene 205** | **Nucleus** | **transcription regulator** |
| **Fos** | **Fos proto-oncogene, AP-1 transcription factor subunit** | **Nucleus** | **transcription regulator** |
| **Socs2** | **suppressor of cytokine signaling 2** | **Cytoplasm** | **other** |
| **C4b*** | **complement C4B (Chido blood group)** | **Extracellular Space** | **peptidase** |
| **Hcn2** | **hyperpolarization activated cyclic nucleotide gated potassium channel 2** | **Plasma Membrane** | **ion channel** |
| **Trp53i11** | **tumor protein p53 inducible protein 11** | **Other** | **other** |
| **Bcl6b** | **B-cell CLL/lymphoma 6B** | **Nucleus** | **transcription regulator** |
| **Acta1*** | **actin, alpha 1, skeletal muscle** | **Cytoplasm** | **other** |
| **Aplnr*** | **apelin receptor** | **Plasma Membrane** | **G-protein coupled receptor** |

***Gene related to cardiac structure or function**
