# Supplementary figures and images for: Cisd2 is essential to delaying cardiac aging and to maintaining heart functions
Source: PLoS Biol. 2019 Oct 8;17(10):e3000508. doi: 10.1371/journal.pbio.3000508 (PMC6799937; doi:10.1371/journal.pbio.3000508)

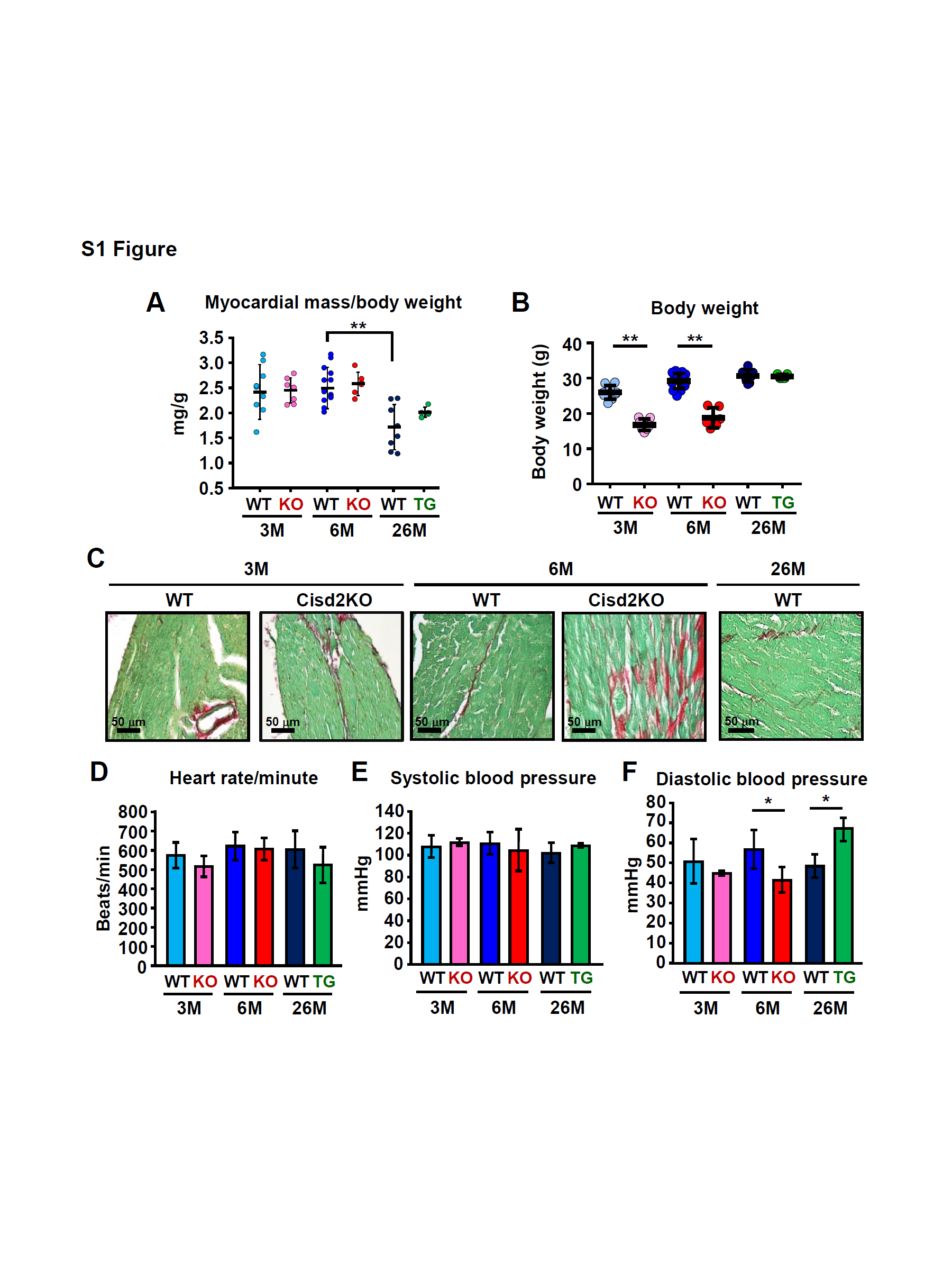

Supplement: S1 Fig — Related to Figs 1 and 2. The heart weight (A) and body weight (B) were measured at the time of euthanasia. (C) High-power section of the papillary muscle with Sirius Red/Fast Green staining of Fig 1D. An increase in the collagen fibers present is notable in the old WT mice at 26M and in the Cisd2KO mice at 3M and 6M. Scale bar, 50 μm. Heart rate (D) was measured from 5 minutes of sequential beats of whole ECG tracings. Systolic blood pressure (E) and diastolic blood pressure (F) of WT, Cisd2KO, and Cisd2TG were measured from conscious mice at designated ages using the noninvasive tail-cuffs BP-2000 Blood Pressure Analysis System (Visitech Systems, Apex, NC, USA). Blood pressure values were recorded 20 times in rapid succession, and the mean value was generated for each individual mouse. The data are presented as mean ± SD. *p < 0.05; **p < 0.005. Values for each data point can be found in S1 Data. 3M, 3 months old; 6M, 6 months old; 26M, 26 months old; Cisd2KO, CDGSH iron-sulfur domain-containing protein 2 knockout; Cisd2TG, CDGSH iron-sulfur domain-containing protein 2 transgenic; ECG, electrocardiography; WT, wild type. (TIF) [file pbio.3000508.s004.tif]

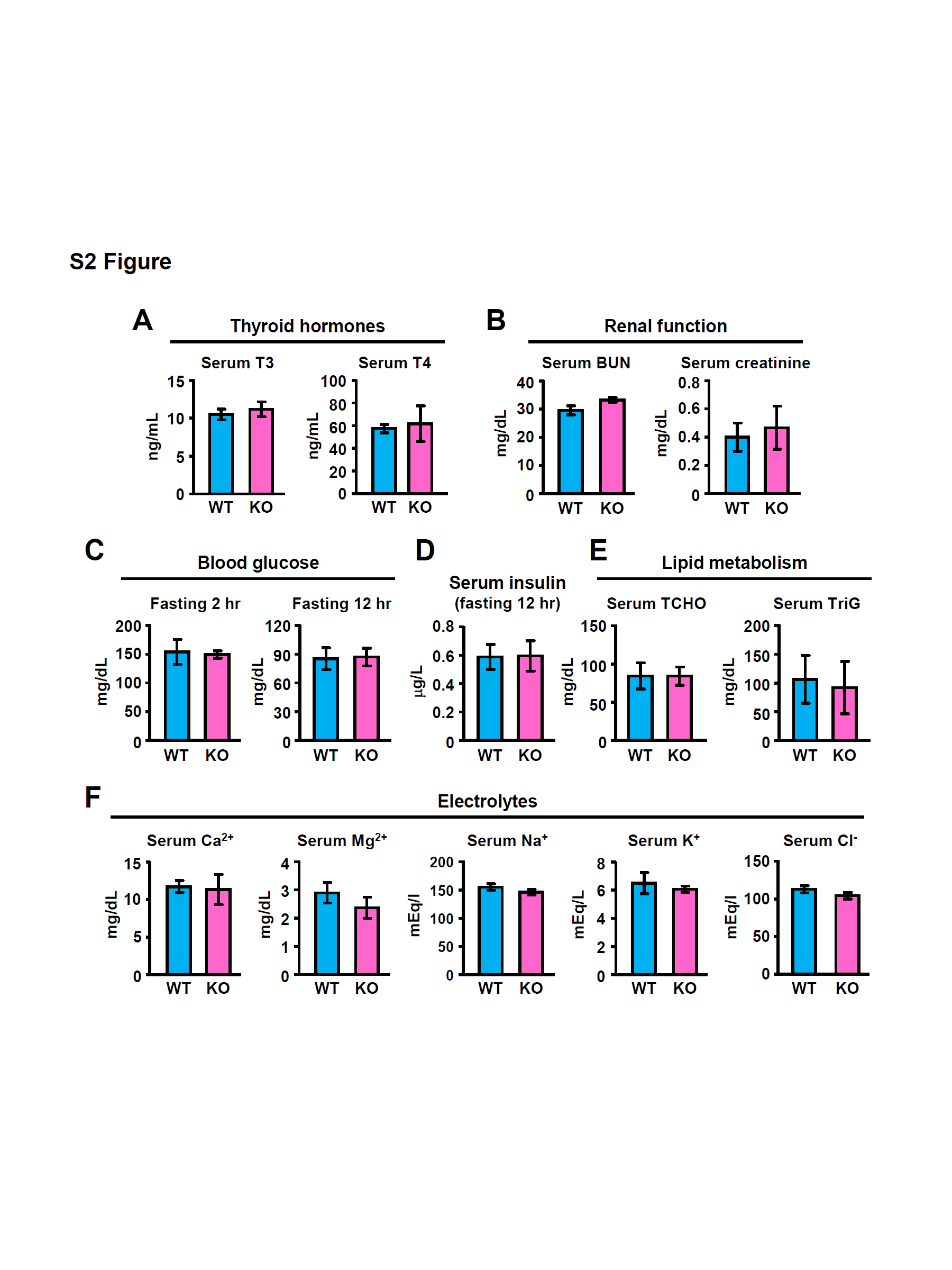

Supplement: S2 Fig — Related to Fig 2. (A) Serum T3 and T4 levels (mouse number n = 3). (B) Serum BUN and creatinine levels (mouse number n = 3). (C) Blood glucose levels after 2 and 12 hours of fasting (mouse number n = 4–8). (D) Serum insulin levels after 12 hours of fasting (mouse number n = 3–5). (E) Serum TCHO and TriG levels (mouse number n = 7–8). (F) Serum Ca2+, Mg2+, Na+, K+, and Cl− levels (mouse number n = 3). The data are presented as mean ± SD. *p < 0.05; **p < 0.005. Values for each data point can be found in S1 Data. BUN, blood urea nitrogen; Cisd2KO, CDGSH iron-sulfur domain-containing protein 2 knockout; T3, triiodothyronine; T4, thyroxine; TCHO, total cholesterol; TriG, triacylglycerol; WT, wild type. (TIF) [file pbio.3000508.s005.tif]

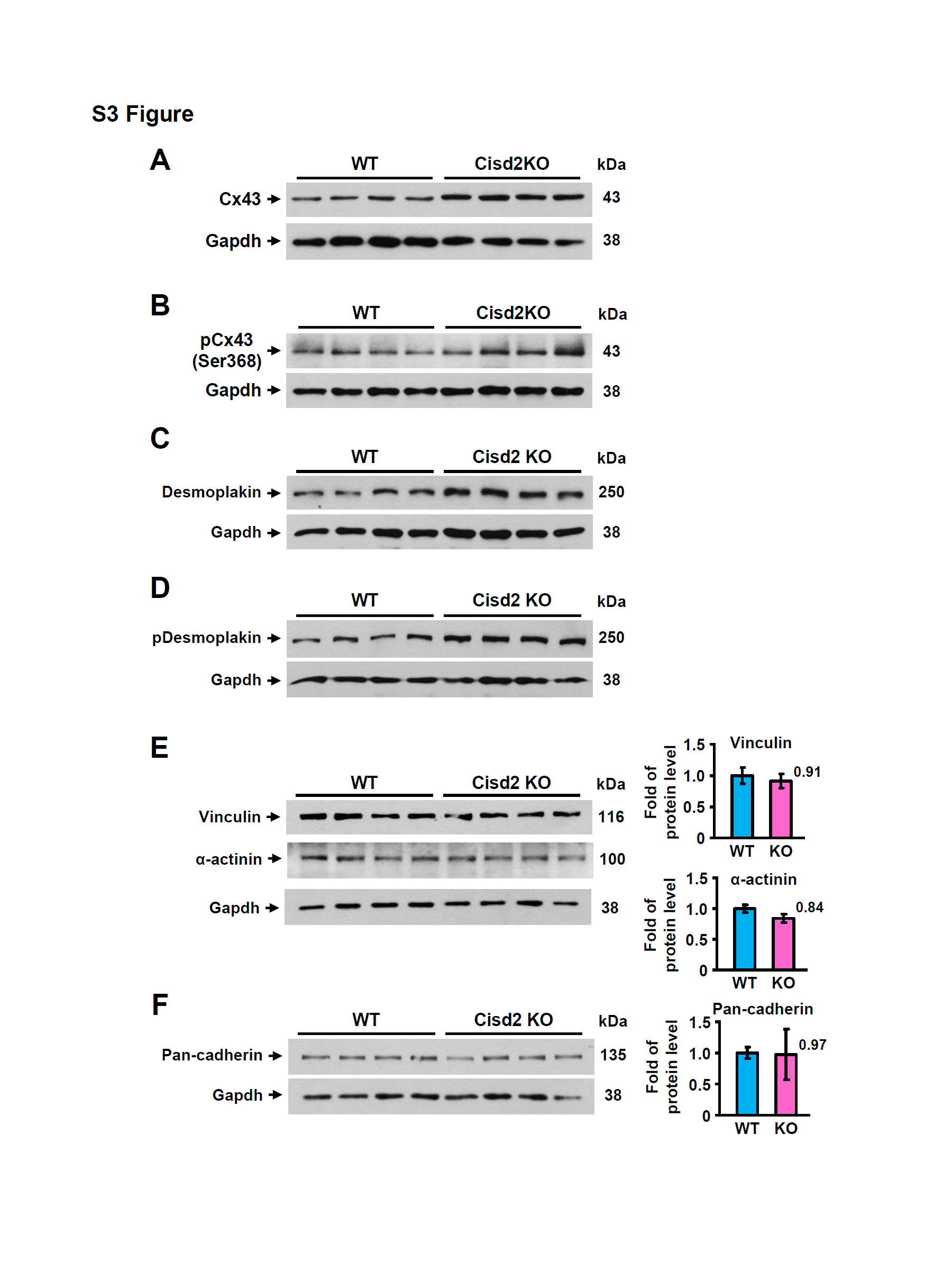

Supplement: S3 Fig — Related to Fig 3. Western blot analyses of Cx43 (A), phosphorylated form of Cx43 (Ser368) (B), desmoplakin (C), phosphorylated form of desmoplakin (D), vinculin and α-actinin (E), and pan-cadherin (F) for the heart tissues of WT and Cisd2KO mice at 3 months old. There are four animals for each group of mice. The data are presented as mean ± SD. *p < 0.05; **p < 0.005. Values for each data point can be found in S1 Data. Cisd2KO, CDGSH iron-sulfur domain-containing protein 2 knockout; Cx43, Connexin 43; WT, wild type. (TIF) [file pbio.3000508.s006.tif]

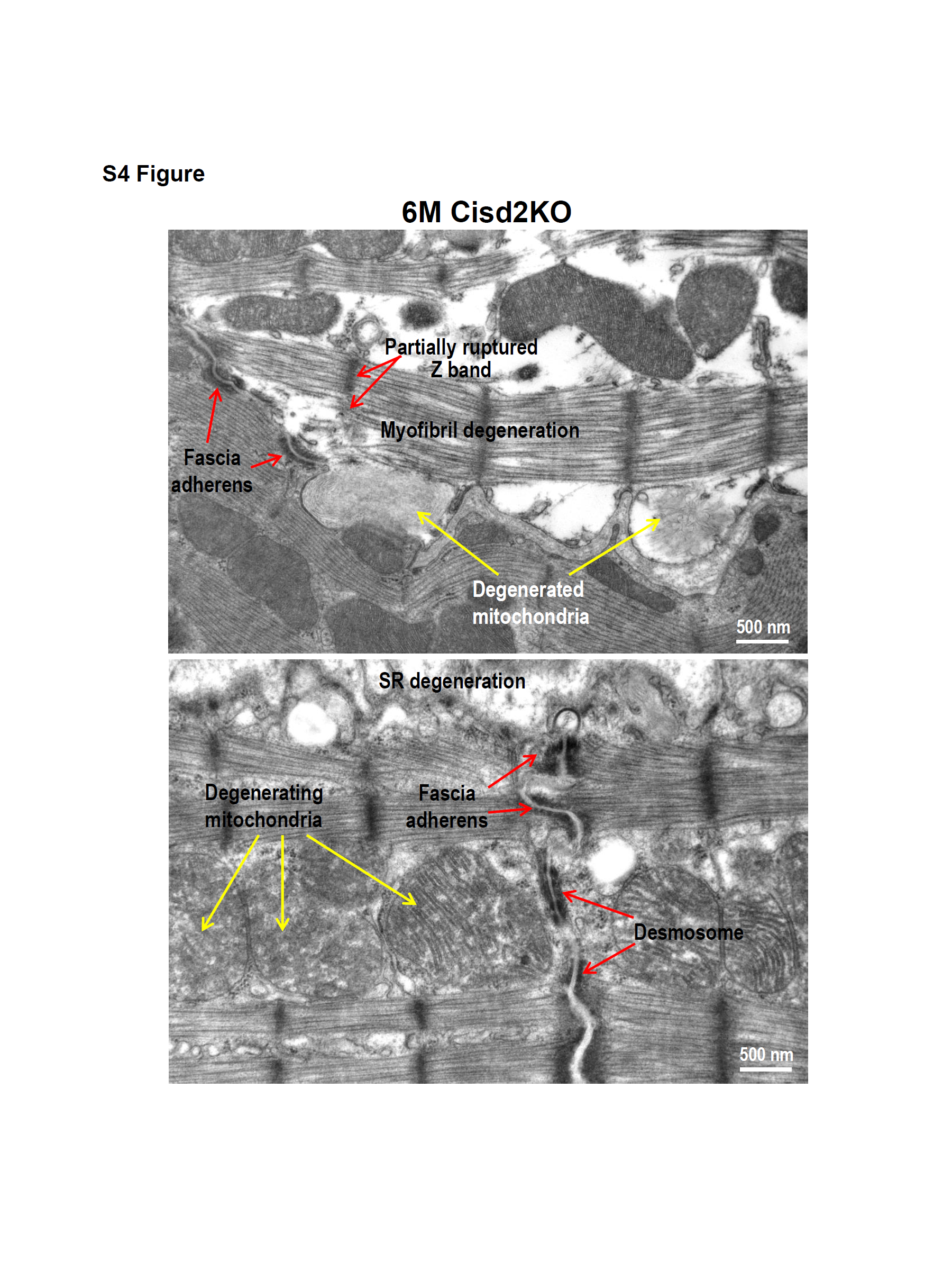

Supplement: S4 Fig — Related to Fig 3. The two representative TEM micrographs revealed severe ultrastructure defects in the cardiac muscle of 6M Cisd2KO mice. Notably, SR degeneration with dilated cisternae and myofibril degeneration with a decreased number of myofibrils, as well as partial disruption of some Z bands, were more severe and easily detected at 6 months of age. Moreover, the severity of the mitochondrial damages—including mitochondria with ruptured outer and inner membranes, swollen mitochondria, and pale mitochondria with fewer cristae—is also more obvious in the Cisd2KO heart at 6M. 6M, 6 months old; Cisd2KO, CDGSH iron-sulfur domain-containing protein 2 knockout; SR, sarcoplasmic reticulum; TEM, transmission electron microscopy. (TIF) [file pbio.3000508.s007.tif]

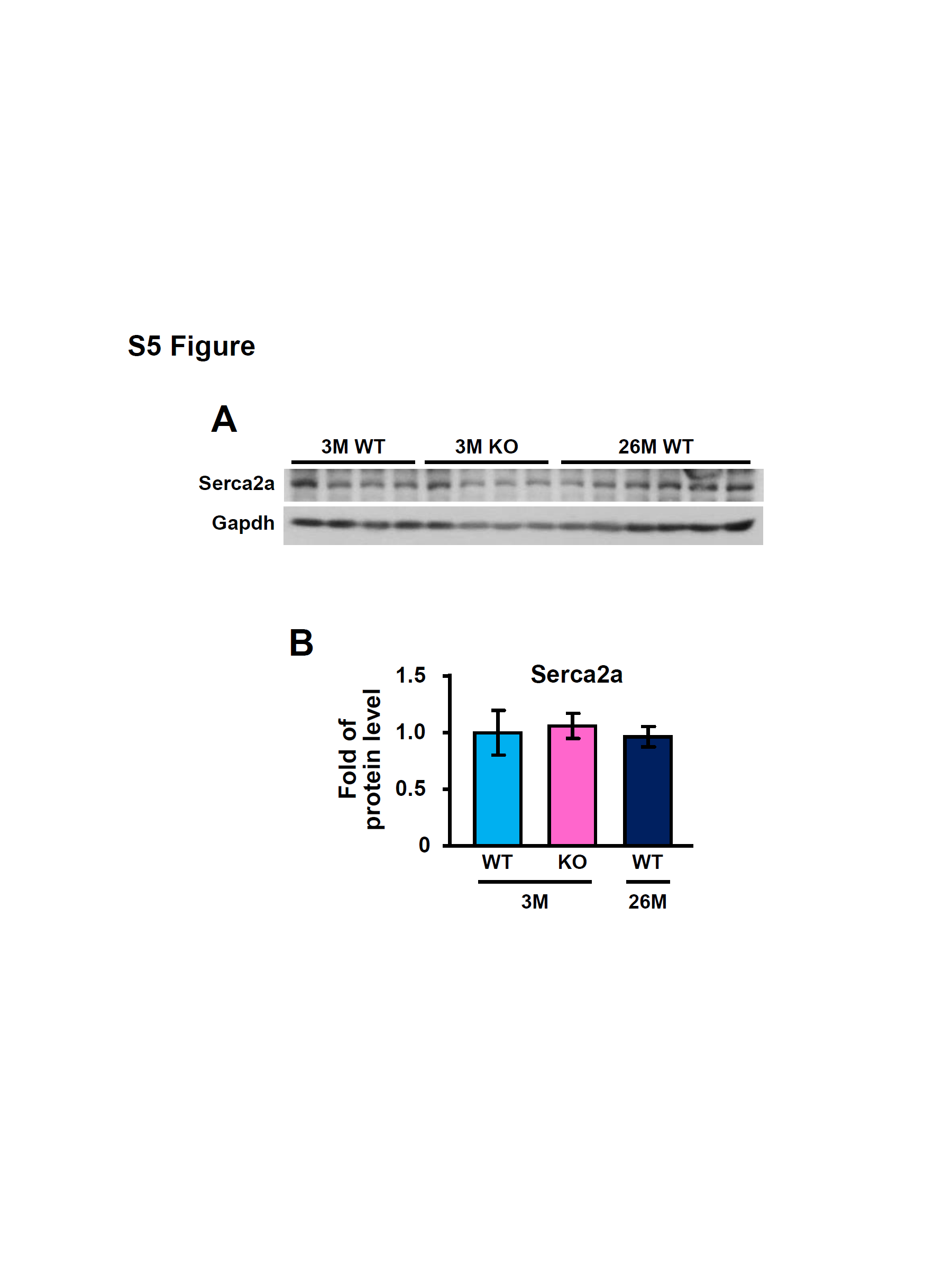

Supplement: S5 Fig — Related to Fig 4. (A and B) Western blot analysis (A) and quantification (B) of Serca2a protein levels in the hearts of WT (n = 4), Cisd2KO (n = 4), and aged WT mice (n = 6). The data are presented as mean ± SD. Values for each data point can be found in S1 Data. Cisd2KO, CDGSH iron-sulfur domain-containing protein 2 knockout; Serca2a, sarco/endoplasmic reticulum Ca2+-ATPase; WT, wild type. (TIF) [file pbio.3000508.s008.tif]

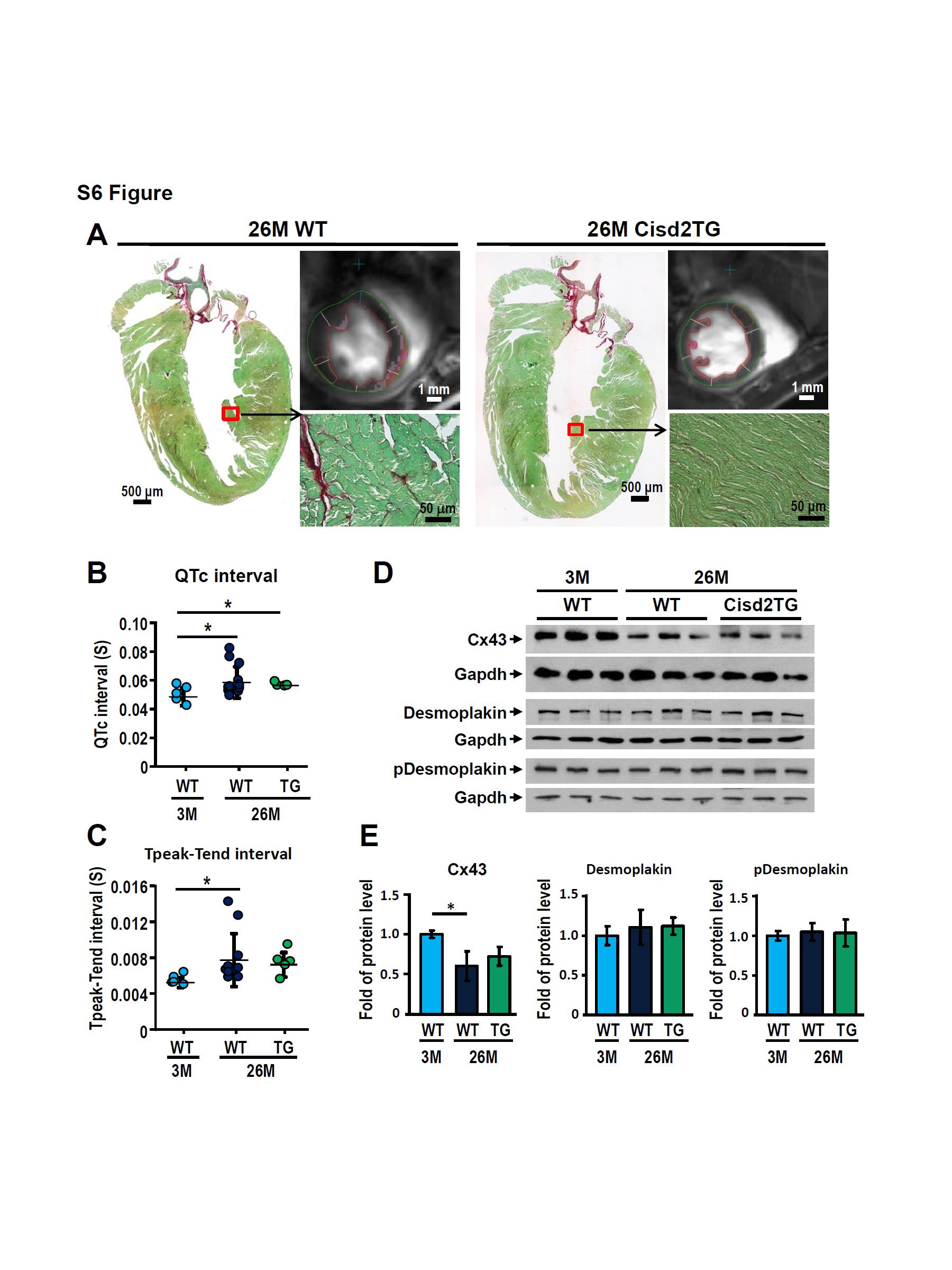

Supplement: S6 Fig — Related to Fig 5. (A) Long-axis low-power section of heart and high-power section of papillary muscle stained with Sirius Red/Fast Green for detection of collagen. Representative cardiac MRI is also shown. (B) Corrected QT interval measurements made from 5-minute sequential beats of whole ECG tracings from baseline. (C) Tpeak–Tend interval measurements made from 5 minutes of sequential beats of whole ECG tracings from baseline. (D and E) Western blot analysis (D) and quantification (E) of protein levels of Cx43, desmoplakin, and phosphorylated desmoplakin in the hearts of WT and Cisd2TG mice (n = 3). The data are presented as mean ± SD. *p < 0.05. Values for each data point can be found in S1 Data. 26M, 26 months old; Cisd2TG, CDGSH iron-sulfur domain-containing protein 2 transgenic; Cx43, Connexin 43; ECG, electrocardiography; MRI, magnetic resonance imaging; WT, wild type. (TIF) [file pbio.3000508.s009.tif]

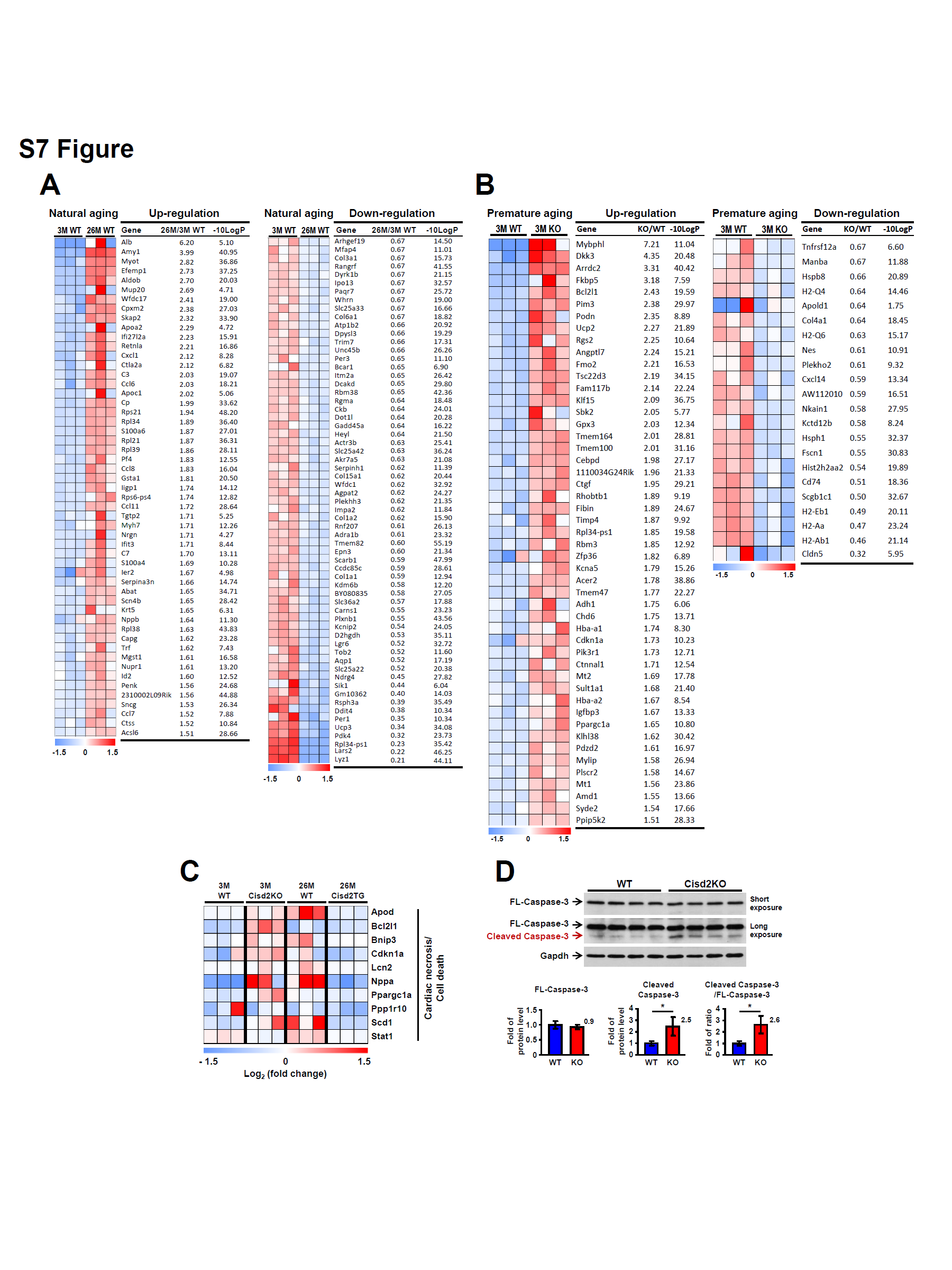

Supplement: S7 Fig — Related to Fig 6. (A) List of 116 differentially expressed mRNAs (up 53 + down 63) uniquely identified in the hearts of naturally aged mice (26M WT versus 3M WT). (B) List of 71 differentially expressed mRNAs (up 49 + down 22) uniquely identified in the hearts of prematurely aged mice (3M Cisd2KO versus 3M WT). (C) Heatmap illustrating the mRNA levels of genes related to cardiac necrosis/cell death pathway by IPA classification. The mRNA expression level was analyzed by RNA sequencing. (D) Western blot analyses and quantification of FL-Caspase-3 and cleaved Caspase-3 in the hearts of WT and Cisd2KO mice at 6 months old (n = 4). The data are presented as mean ± SD. *p < 0.05. Values for each data point can be found in S1 Data. 3M, 3 months old; 26M, 26 months old; Cisd2KO, CDGSH iron-sulfur domain-containing protein 2 knockout; DEG, differentially expressed gene; FL, full-length; IPA, Ingenuity Pathway Analysis; WT, wild type. (TIF) [file pbio.3000508.s010.tif]

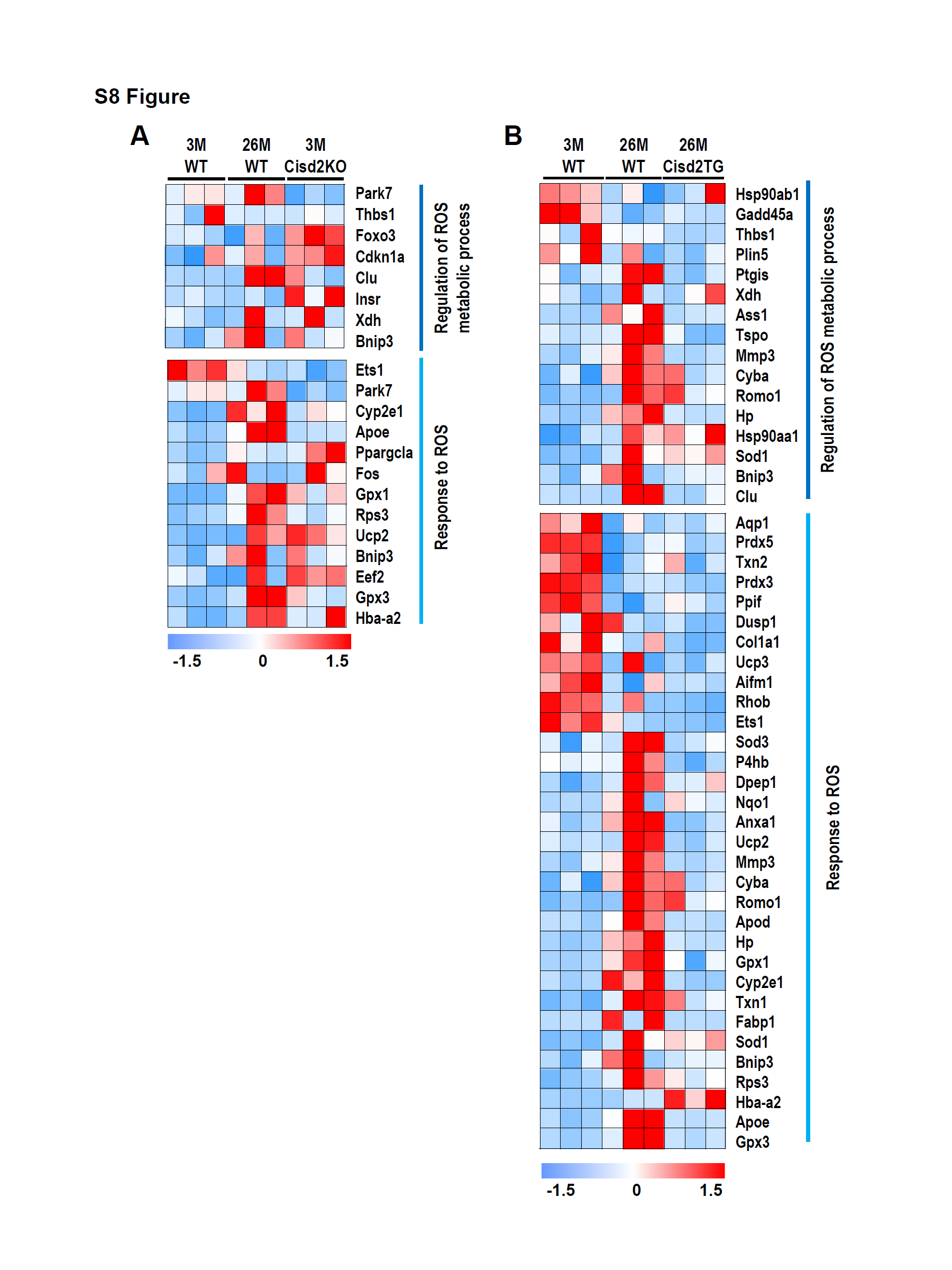

Supplement: S8 Fig — Related to Fig 6. (A) Heatmap illustrating the DEGs identified in the hearts of naturally aged mice (26M WT) and prematurely aged mice (3M Cisd2KO) compared with young mice (3M WT). (B) Heatmap illustrating the DEGs identified in the hearts of naturally aged mice (26M WT) and long-lived mice (26M Cisd2TG) compared with young mice (3M WT). Values for each data point can be found in S1 Data. 3M, 3 months old; 26M, 26 months old; Cisd2KO, CDGSH iron-sulfur domain-containing protein 2 knockout; Cisd2TG, CDGSH iron-sulfur domain-containing protein 2 transgenic; DEG, differentially expressed gene; IPA, Ingenuity Pathway Analysis; ROS, reactive oxygen species; WT, wild type. (TIF) [file pbio.3000508.s011.tif]

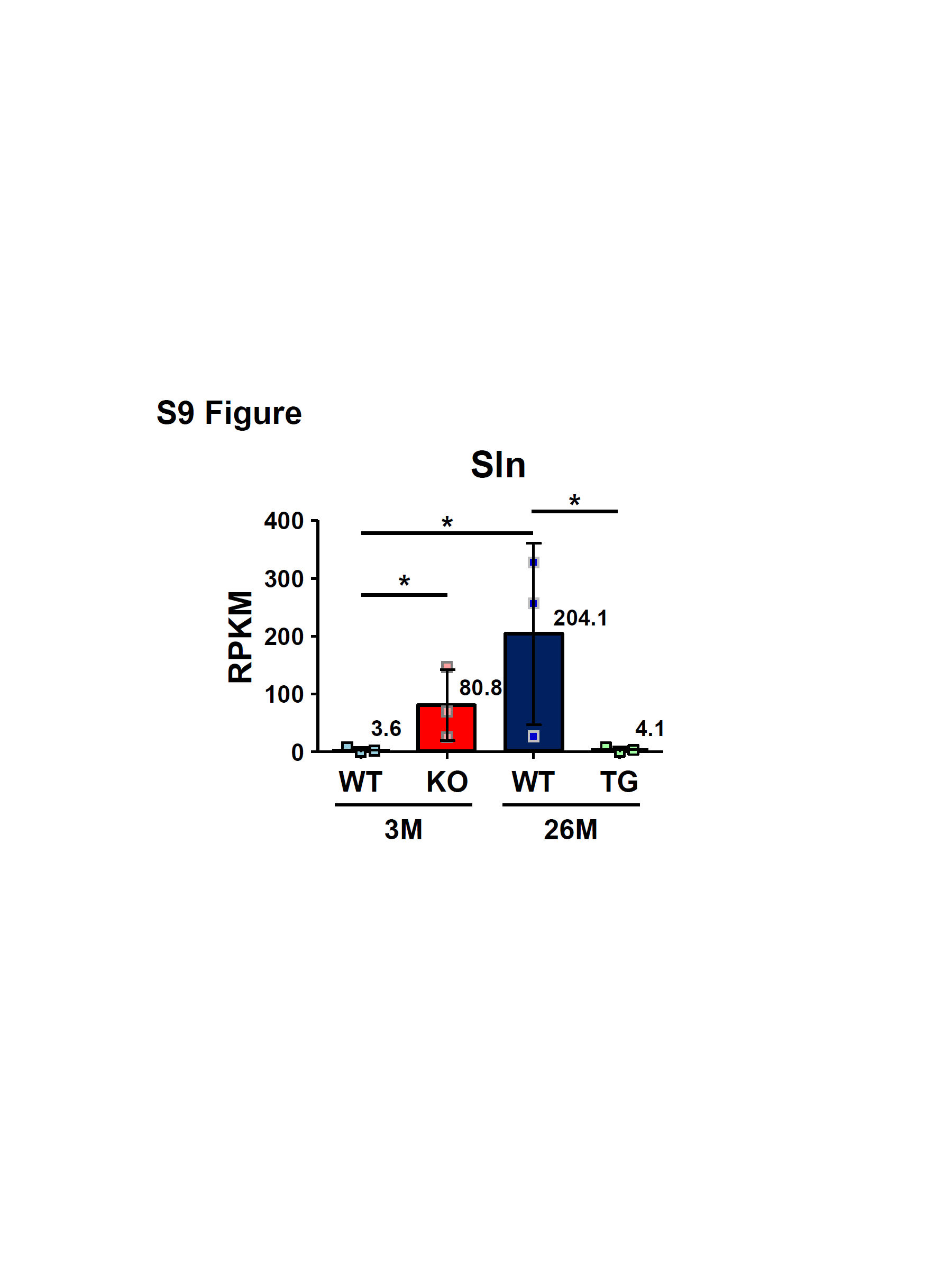

Supplement: S9 Fig — Related to Fig 6 and discussion. The Sln mRNA levels were obtained from a transcriptomics analysis using RNA sequencing. The data are presented as mean ± SD. *p < 0.05 by one-tailed t test. Values for each data point can be found in S1 Data. Cisd2KO, CDGSH iron-sulfur domain-containing protein 2 knockout; Cisd2TG, CDGSH iron-sulfur domain-containing protein 2 transgenic; RPKM, reads per kilobase of exon model per million reads; Sln, sarcolipin; WT, wild type. (TIF) [file pbio.3000508.s012.tif]
