# Supplementary material for: Cisd2 is essential to delaying cardiac aging and to maintaining heart functions
Source: PLoS Biol. 2019 Oct 8;17(10):e3000508. doi: 10.1371/journal.pbio.3000508 (PMC6799937; doi:10.1371/journal.pbio.3000508)
Supplement: S1 Text — (DOCX) [file pbio.3000508.s019.docx]

**Supporting Information**

**Detail methods**

**EXPERIMENTAL MODEL AND SUBJECT DETAILS**

**Animal Care**

Cisd2KO [1] and Cisd2TG [2] mice were generated as previously described. Male mice were used for all experiments. All mouse lines have a pure or congenic C57BL/6 background and were bred/housed in a specific pathogen-free facility with a 12 hr light/12hr dark cycle at constant temperature (23°C) and allowed free access to food and water. The animal protocols were approved by the Institutional Animal Care and Use Committee of Chang Gung Memorial Hospital and National Yang-Ming University.

**Cardiac MRI**

Cardiac MRI (ClinScan 70/30, Bruker, Germany) were acquired under isoflurane inhalation anesthesia with ECG and respiration gating applied in conjunction voiding cardiac and chest motion which might spoil the image quality. CMR protocol in this case is arranged as follows [3-5]. A three-segment localizer was first performed for anatomical reference, which could be adjusted to short-axis, long-axis, and four-chamber coordination for following examinations. Short-axis CINE scans with ECG and respiration gating were acquired to record various time frames including end-systole and end-diastole phases. A FLASH sequence was adapted with TR/TE 15/1.67ms, slice thickness 0.7 mm, flip angle 15°, field of view 45x45 mm^2^, average 3, and in-plane resolution 0.243 x 0.182 mm^2^. Moreover, multiple slices were placed evenly from base to apex to cover the whole heart. Short-axis delayed enhancement imaging was initiated at 10 min post a 0.2 mmol/kg bolus injection of Gd-DTPA (Magnevist®, Bayer) via intraperitoneal route. Specific parameters are TR/TE 422.5/1.31ms, slice thickness 1.5mm, flip angle 15°, average 20, inversion time 310ms, field of view 35x35 mm^2^, and in-plane resolution 0.243 x 0.182 mm^2^. Subsequently, CMR images were loaded to QMass (Medis, The Netherlands) for further quantitative analysis [6], including automatic/manual delineation of myocardium, left ventricular volume, ejection fraction, wall motion, and wall thickness scoring. The different segments explore the effects of aging on the cardiac muscles from each of the territories supplied by the three major coronary arteries. Diastolic phase (Segment 7+8): The middle anterior (segment 7) and middle anteroseptal (segment 8) are supplied by left anterior descending coronary artery. Diastolic phase (Segment 10+11): the middle inferior (segment 10) and middle inferolateral (segment 11) are supplied by right coronary artery and left circumflex artery, respectively.

**In vivo pressure-volume analysis**

Pressure-volume analysis was performed according to established open-chest retrograde insertion method [7]. Each mouse was anesthetized (induction: 3% isoflurane, maintenance: 1%–2% isoflurane), intubated, and mechanically ventilated (MiniVent, model 845, Harvard Apparatus) with 100% O_2_. The animal was then placed on a temperature-controlled heating platform to maintain body temperature. The diaphragm and ribs were dissected, the LV apex was punctured with a 27-gauge needle followed with insertion of the Millar catheter (Millar 1.4F, SPR 839, Millar Instruments). Mice were allowed to stabilize for 5 minutes, after which transient occlusion of inferior vena cava was performed.

**Electrocardiography**

The mice were maintained on a 12:12 hour dark-light cycle with lights switched on at 6:00 am. All procedures took place during the light phase. Anesthesia was initially induced by placing the mice for 3-5 minutes in a chamber filled with 3% volume-to-volume isoflurane (Aesica Pharmaceuticals, Hertfordshire, UK). The mice were then positioned on a warm pad (ALA Scientific Instruments, Inc. NY) that enabled maintaining temperature during ECG recording. Mice breathed freely through a nose cone. Anesthesia was maintained by inhalation of 1.5% isoflurane. Continuous 5-minute ECGs were obtained using subcutaneous electrodes attached at the four limbs and recorded with PowerLab data acquisition system (model ML866, ADInstruments, Colorado Springs, CO) and Animal Bio Amp (model ML136, ADInstruments). ECG analysis was performed in an unbiased fashion where 1500 beats were analysed using LabChart 7 Pro version 7.3.1 (ADInstruments, Inc). Detection and analysis of QTc interval, QRS intervals, Tpeak-T end intervals were set to Mouse ECG parameters [8]. The values obtained were compared statistically by utilizing the Mann-Whitney U test, and p < 0.05 was accepted as significant.

**Split YFP Assay**

The Split YFP assay was performed as previously described [2]. The cDNA encoding the N-terminus (VN) or C-terminus of Venus (VC) proteins were introduced before the 5′ and after the 3′ of the open reading frame of Cisd2 in order to generate the four fusion proteins, VN-Cisd2, Cisd2-VN, VC-Cisd2 and Cisd2-VC. Similarly, cDNA encoding the VN or VC proteins were introduced before the 5′ and after the 3′ of the open reading frame of Serca2a in order to generate the four fusion proteins, VN-Serca2a, Serca2a-VN, VC-Serca2a and Serca2a-VC. Different combinations of the constructs were co-transfected into HEK293T cells for 24 hr. To monitor the direct interaction between Cisd2 and Serca2a, fluorescence images of the cells were captured using a confocal microscope (Zeiss LSM 700).

**Primary Adult Cardiomyocyte Preparation**

Primary adult mouse cardiomyocytes were isolated from 8- to 10-weeks-old male WT, Cisd2KO and Cisd2TG mice following Langendorf perfusion as described [9]. Rod-shaped cardiomyocytes with clear striations were considered healthy cells and were chosen for Ca^2+^ imaging.

**Spontaneous Ca^2+^ waves detection**

A LeicaTCS SP8x scanning system (Leica Microsystems, Wetzlar, Germany) equipped with a × 63 oil immersion objective was used for confocal imaging of Ca^2+^ fluorescence with line scan mode. Fluo-4 AM was excited by white light laser at 490 nm and emission signals was collected over 500nm~560nm. Digital image processing was performed by using Las X Ver 3.5.5 (Leica Microsystems, Wetzlar, Germany) and MetaMorph Ver 7.10 (Molecular Devices, LLC., San Jose, CA, USA). The Ca^2+^ level was reported as F1/F0 (or as ΔF /F0), where F0 was the resting Ca^2+^ fluorescence.

**Measurement of Intracellular Calcium Levels Using Fura-2/AM**

The cytosolic Ca^2+^ levels of single cells were measured as previously described [10]. Briefly, the primary isolated adult cardiomyocytes on a fibronectin/gelatin-coated coverslip were stained with 5 μM Fura-2/AM in loading buffer (150 mM NaCl, 5 mM glucose, 10 mM HEPES, 1 mM MgCl_2_, 5 mM KCl and 2 mM CaCl_2_ at pH 7.4) for 60 min at 37^o^C in the dark. After washing the adult cardiomyocytes twice by loading buffer, the coverslip was transferred to an examination chamber containing 1 ml fresh loading buffer, and the cytosolic Ca^2+^ concentration was measured using the fluorescence intensity ratio of Ca^2+^-bound Fura-2 to unbound Fura-2 excited at 340 and 380 nm, respectively, with emission measured at 510 nm and these measurements were carried out at 2 s intervals. The switching excitation wavelengths were provided by a monochromator (Polychrome V, TILL Photonics GmbH Gräfelfing, Germany), and the fluorescence images were acquired through a CCD camera (Micromax YHS1300, Roper Scientific, Trenton, NJ, U.S.A.) attached to a fluorescence microscope (IX70, Olympus, Tokyo, Japan). The monochromator, CCD camera, and image acquisition were controlled by MetaFluor (Molecular Devices, Downingtown, PA, U.S.A.). [Ca^2+^]i was calculated using a Kd of 224 nM as previously described [11]. ER or mitochondrial Ca2+ store were quantified following 2 μM thapsigargin (Tag)-induced ER Ca^2+^ release or 2 μM of uncoupling agent carbonyl cyanide m-chlorophenyl hydrazone (CCCP)-induced mitochondrial Ca^2+^ release, respectively. The basal and maximum fluorescence intensities were recorded for each adult cardiomyocyte during the experiment. Maximal Ca^2+^ release from ER or mitochondria were determined by the difference between basal and peak [Ca^2+^]i evoked by Tag or CCCP, respectively.

**Cardiac Tissue Reactive Oxygen Species (ROS) and Reactive Nitrogen Species (RNS) Levels**

ROS and RNS levels were detected from cardiac tissue lysate using an in vitro ROS/RNS Assay Kit assay kit for ROS and RNS quantitation according to the manufacturer’s instructions (STA-347, Cell Biolabs). The fluorescence intensity of 2’,7’-dichlorodihydrofluorescein (DCF) was measured using an Infinite 200 Microplate Reader (Tecan Group Ltd).

**Mitochondrial membrane potential determined by JC-1 assay, confocal microscopy, and flow cytometry**

The mitochondria membrane potential was measured by JC-1 staining (T3168, Thermo). For the confocal microscopy, the primary isolated adult cardiomyocytes were cultured on a fibronectin/gelatin-coated µ-Dish 35 mm (81156, ibidi) and stained with 5 μM JC-1 dye in culture medium for 30 minutes at 37^o^C in the dark. The adult cardiomyocytes were washed by culture medium in the µ-Dish and imaged by ZEISS LSM 700 (ZEISS). For the flow cytometry, the primary isolated adult cardiomyocytes were stained with 5 μM JC-1 dye in culture medium for 30 minutes at 37^o^C in the dark. The stained adult cardiomyocytes were acquired using FACSCanto (BD Biosciences) and analyzed by Flowjo (FlowJo, LLC).

**Mitochondrial DNA Deletion**

The mitochondrial D-1 and D-17 deletions were detected by genomic DNA isolated from heart tissues and then PCR amplified of the mitochondrial DNA as described previously [12].

**Serca2a Activity Assay**

The Serca2a activity was measured in total heart tissue lysate using an enzyme-coupled spectrophotometric assay [13,14]. Briefly, the heart tissues were homogenized in ice-cold homogenization buffer (0.25 M sucrose, 5 mM HEPES, 0.2% NaN_3_ and protease inhibitor cocktail [P8340, Sigma] at pH 7.3). The heart homogenate was added as 100 μg/ml of protein into 1 ml of reaction buffer (200 mM KCl, 20 mM HEPES, 15 mM MgCl_2_, 10 mM NaN_3_, 10 mM Phosphoenolpyruvate, 5 mM ATP, 1 mM EGTA at pH 7.3) with 1 μM calcimycin A-23187 and 1 mM CaCl_2_ for 3 min at 37^o^C. Immediately before measurement, 18 U/ml lactate dehydrogenase (LDH), 12 U/ml pyruvate kinase (PK), 0.5 mM NADH and lystae/reaction buffer mixture were added into a plastic cuvette. After the reagents were added into the cuvette, the absorbance at 340 nm was measured using a UV/visible spectrophotometer at 37^o^C. Serca2a-independent Ca^2+^-ATPase activity was determined in the presence of the Serca specific inhibitor 2’,5’-di(tert-butyl)-1,4-benzohydroquinone (TBQ) (40 μM). The level of oxidized NADH (340 nm) was calculated by the absorption coefficient of NADH (6.22 mM^-1^ x cm^-1^). The activity of ATPase was calculated as the oxidized NADH level (nmole) normalized to reaction time (min) and protein content (mg).

**Subcellular fractionation of heart tissues**

Fresh heart tissues were washed twice with PBS and homogenized immediately in ice-cold SEH buffer (0.25 M sucrose, 1 mM EGTA, 3 mM HEPES and protease inhibitor cocktail at pH 7.2) by Dounce tissue grinder (357542, Wheaton) on ice. After tissue had been homogenized, the cell debris and nuclei were removed by low-speed centrifugation (800g), and then transfer the homogenate (H) to a new eppendorf. The crude cytosolic (Crude Cyto.) fraction and crude mitochondrial (Crude Mito.) fraction were obtained by high-speed centrifugation (10,000g) of the homogenate as the supernatant and pellet, respectively. The pure cytosolic (Pure Cyto.) fraction and microsome pellet (SR) were obtained by ultrahigh-speed centrifugation (100,000g) of the crude cytosolic fraction for 1 hr at 4^o^C as the supernatant and pellet, respectively. The mitochondrial and SR pellets were suspended by SEH buffer and then for western blot analysis.

**Measurement of Mitochondrial Oxygen Consumption Rate Using a Seahorse Analyzer**

An XF^e^24 analyzer (Seahorse Bioscience, MA) was applied to measure the oxygen consumption rate (OCR) of mitochondria. The primary isolated adult cardiomyocytes were cultured in a laminin-coated XF24 V7 plate at 6 x 10^3^ cells/well. The primary adult cardiomyocytes were kept in normal growth medium for 6 hr and then replaced by sodium bicarbonate-free DMEM supplemented with 2% FBS, 4 mM glutamine, 100 U/ml penicillin, 100 μg/ml streptomycin, 1 mM sodium pyruvate and 1% insulin-transferrin-selenium (ITS) at 1 hr before measurement. The OCR was measured before and after added the indicated chemicals (1 μM oligomycin A, 1 μM FCCP and 1 μM rotenone with 1 μM antimycin A) at 37^o^C. The results are presented in pmol/min/μg protein.

**Western Blotting**

Heart tissue samples were homogenized in RIPA buffer (50 mM Tris at pH 7.4, 150 mM NaCl, 1% Triton X-100, 0.5% Sodium deoxycholate, 0.1% SDS with complete protease inhibitor and phosphatase inhibitor cocktails [Roche]) and denatured in 2% SDS sample buffer (50 mM Tris at pH 6.8, 100 mM Dithiothreitol, 2% SDS and 10% glycerol) for 10 min at 100^o^C. The extracted proteins were separated by SDS-polyacrylamide gel electrophoresis (Bio-Rad) and electro-transferred to a polyvinylidene fluoride (PVDF) membrane (PerkinElmer). The membranes were blocked with 5% (w/v) nonfat dry milk, probed with antibody, and detected by an ECL (WBKLS0500, Millipore). The following antibodies were used for western blotting: Cisd2,^1^ Gapdh (MAB374, Millipore), Serca2 (MA3-919, Thermo), Atp5b (A-21351, Thermo), α-actinin (A7811, Sigma), Calnexin (C4731, Sigma). Connexin 43 (C8093, Sigma), p-Connexin 43 (Ser368) (3511, Cell signaling), Pan-Cadherin (C3678, Sigma), Vinculin (V9131, Sigma), Desmoplakin (25318-1-AP, Proteintech), p-Desmoplakin (5885, Cell signaling), Caspase-3 (9662, Cell signaling).

**Serum Biochemical Analysis**

Whole blood was collected from facial vein. Serum total cholesterol (TCHO), triglyceride (TriG), blood urea nitrogen (BUN), creatinine, Ca^2+^, Mg^2+^ and K^+^ levels were analyzed by Fuji Dri-Chem 4000i (FUJIFILM). Serum insulin (10-1247-01, Mercodia), triiodothyronine (T3) (CEA453Ge, Cloud-Clone Corp.) and thyroxine (T4) (CEA452Ge, Cloud-Clone Corp.) were analyzed by ELISA kits.

**Histology**

Mouse hearts were collected, fixed with 10% formalin buffered with phosphate, underwent tissue processing and embedded in paraffin. Sirius Red staining (Muto Pure Chemicals) of tissue sections (3-4 μm) were carried out using standard protocols.

**Immunofluorescence (IF), Confocal Microscopy, and Cx43/pan-cadherin colocalization** **coefficiency**

Immunofluorescent staining was performed on optimal cutting temperature (OCT)-embedded cryosections (16 μm) with the following antibodies: pan-cadherin (C3678, Sigma), Desmoplakin (CBL173, Millipore), Cx43 (C8093, Sigma), Vinculin (V9131, Sigma), and α-actinin (A7811, Sigma). The wheat germ agglutinin (WGA) or α-actinin co-staining with nucleus staining dye Hoechst (33342, Cell signaling). Gap junction remodeling was quantified by determining the extent of colocalization of Cnx43 and pan-cadherin at the intercalated discs using the Pearson–Spearman correlation colocalization (PSC) plugin for ImageJ [15].

**Transmission Electron Microscopy (TEM)**

Mouse hearts were fixed in a mixture of 1.5% glutaraldehyde and 1.5% paraformaldehyde in 0.1 M cacodylate buffer at pH 7.3. Heart tissues were post-fixed in 1% OsO_4_ and 1.5% potassium hexanoferrate, and then tissues were rinsed in cacodylate and 0.2 M sodium maleate buffers (pH 6.0) followed by block-stained with 1% uranyl acetate. Following dehydration, the hearts were embedded in Epon (14120, EMS) and sectioned for TEM as described previously [16]. Overt ultrastructural abnormalities, namely shortening and breaking-down of the fascia adherens (FA), and degeneration of the mitochondria, including mitochondria with ruptured outer and inner membranes, swollen mitochondria with fewer crista, as well as extension and fragmentation of gap junctions.

**RNA Sequencing and Pathway Analysis**

The RNA sequencing was performed by National Yang-Ming University VYM Genome Research Center. The differentially expressed genes (DEGs) calculated from the RPKM (reads per kilobase of exon model per million reads) in RNA-Seq data were further annotated by the IPA approach (Ingenuity Systems ®, www.ingenuity.com). The functional characterization was conducted by online tools PANTHER ([www.pantherdb.org](http://www.pantherdb.org)) [17] and STRING (<https://string-db.org>) [18]. Heat maps were graphed by loading log-transformed fold changes into Multi Experiment Viewer (MEV) 4.9 software [19].

**Statistical Analysis**

The data are presented as mean ± SD or mean ± SEM, as described in the figure legends. Comparisons between the two groups were done using an unpaired two-tailed Student’s t test. When analyzing statistical differences among groups, *p* < 0.05 was considered significant. Statistical analysis was made by using the software Graphpad Prism 6.0.

**Supplemental References**

1. Chen YF, Kao CH, Chen YT, Wang CH, Wu CY, Tsai CY, et al. Cisd2 deficiency drives premature aging and causes mitochondria-mediated defects in mice. Genes & development. 2009;23:1183-1194.

2. Shen ZQ, Chen YF, Chen JR, Jou YS, Wu PC, Kao CH, et al. CISD2 Haploinsufficiency Disrupts Calcium Homeostasis, Causes Nonalcoholic Fatty Liver Disease, and Promotes Hepatocellular Carcinoma. Cell reports. 2017;21:2198-2211.

3. Ebert SN, Taylor DG, Nguyen HL, Kodack DP, Beyers RJ, Xu Y, et al. Noninvasive tracking of cardiac embryonic stem cells in vivo using magnetic resonance imaging techniques. Stem Cells. 2007;25:2936-2944.

4. Lee WY, Wei HJ, Lin WW, Yeh YC, Hwang SM, Wang JJ, et al. Enhancement of cell retention and functional benefits in myocardial infarction using human amniotic-fluid stem-cell bodies enriched with endogenous ECM. Biomaterials. 2011;32:5558-5567.

5. Price AN, Cheung KK, Cleary JO, Campbell AE, Riegler J, Lythgoe MF. Cardiovascular Magnetic Resonance Imaging in Experimental Models. The Open Cardiovascular Medicine Journal. 2010;4:278-292.

6. Attili AK, Schuster A, Nagel E, Reiber JH, van der Geest RJ. Quantification in cardiac MRI: advances in image acquisition and processing. The international journal of cardiovascular imaging. 2010;26 Suppl 1:27-40.

7. Pacher P, Nagayama T, Mukhopadhyay P, Batkai S, Kass DA. Measurement of cardiac function using pressure-volume conductance catheter technique in mice and rats. Nat Protoc. 2008;3:1422-1434.

8. Constantinides C, Mean R, Janssen BJ. Effects of isoflurane anesthesia on the cardiovascular function of the C57BL/6 mouse. ILAR journal. 2011;52:e21-e31.

9. Liao R, Jain M. Isolation, culture, and functional analysis of adult mouse cardiomyocytes. Methods Mol Med. 2007;139:251-262.

10. Wu PC, Kao LS. Calcium regulation in mouse mesencephalic neurons-Differential roles of Na(+)/Ca(2+) exchanger, mitochondria and endoplasmic reticulum. Cell calcium 2016;59:299-311.

11. Grynkiewicz G, Poenie M, Tsien RY. A new generation of Ca2+ indicators with greatly improved fluorescence properties. J Biol Chem 1985;260:3440-3450.

12 Tanhauser SM, Laipis PJ. Multiple deletions are detectable in mitochondrial DNA of aging mice. J Biol Chem 1995;270:24769-24775.

13. Gehrig SM, van der Poel C, Sayer TA, Schertzer JD, Henstridge DC, Church JE, et al. Hsp72 preserves muscle function and slows progression of severe muscular dystrophy. Nature. 2012;484:394-398.

14. Simonides WS, van Hardeveld C. An assay for sarcoplasmic reticulum Ca2(+)-ATPase activity in muscle homogenates. Analytical biochemistry. 1990;191:321-331.

15 French AP, Mills S, Swarup R, Bennett MJ, Pridmore TP. Colocalization of fluorescent markers in confocal microscope images of plant cells. Nat Protoc. 2008;3:619-628.

16. Kao CH, Chen JK, Kuo JS, Yang VC. Visualization of the transport pathways of low density lipoproteins across the endothelial cells in the branched regions of rat arteries. Atherosclerosis. 1995;116:27-41.

17. Mi H, Huang X, Muruganujan A, Tang H, Mills C, Kang D, et al. PANTHER version 11: expanded annotation data from Gene Ontology and Reactome pathways, and data analysis tool enhancements. Nucleic acids research. 2017;45:D183-D189.

18. Szklarczyk D, Morris JH, Cook H, Kuhn M, Wyder S, Simonovic M, et al. The STRING database in 2017: quality-controlled protein-protein association networks, made broadly accessible. Nucleic acids research. 2017;45:D362-D368.

19 Saeed AI, Sharov V, White J, Li J, Liang W, Bhagabati N, et al. TM4: a free, open-source system for microarray data management and analysis. Biotechniques. 2003;34:374-837.
